# Generative AI and Large Language Models in Rehabilitation: A Scoping Review

**DOI:** 10.3390/life16081310

**Published:** 2026-08-10

**Authors:** Su-Min Cha

**Affiliations:** Department of Occupational Therapy, Kyungnam University, Changwon 51767, Republic of Korea; csm1206@kyungnam.ac.kr; Tel.: +82-55-249-6479

**Keywords:** generative artificial intelligence, large language models, rehabilitation, clinical reasoning, clinical decision support, rehabilitation planning, prompt engineering

## Abstract

Generative artificial intelligence (AI) and large language models (LLMs) are increasingly evaluated in rehabilitation, yet their clinical validity, reproducibility, and safety remain uncertain. This scoping review mapped peer-reviewed studies of generative AI/LLMs across rehabilitation assessment, clinical reasoning, decision support, planning, education, and functional classification. Following JBI methodology and PRISMA-ScR, five databases were searched for English-language studies published from 1 January 2015 to 24 July 2026. Two reviewers independently conducted study selection, data extraction, methodological appraisal, and application-domain coding. Of 2126 records, 43 publications representing 42 unique studies were included, predominantly from 2025–2026 and involving GPT/ChatGPT/OpenAI-family systems. At the unique-study level, six application domains were identified: clinical reasoning and decision support (*n* = 13), rehabilitation education, simulation, and feedback (*n* = 10), rehabilitation planning and prescription (*n* = 9), guideline adherence and clinical-question support (*n* = 6), adaptive feedback and rehabilitation support (*n* = 2), and assessment and functional classification (*n* = 2). Evidence was concentrated in benchmark, scenario-based, and educational evaluations, with limited patient-level outcomes. Heterogeneous methods, incomplete reporting of reproducibility, inconsistent safety assessment, and possible selective publication limited comparability and clinical generalizability. Generative AI/LLMs should therefore be used primarily as clinician-supervised assistive tools, with prospective validation, standardized reporting, and active safety evaluation prioritized.

## 1. Introduction

Generative artificial intelligence (generative AI) and large language models (LLMs) have rapidly expanded beyond general text generation into healthcare and rehabilitation applications. Conversational systems such as ChatGPT, Gemini, Claude, DeepSeek, Copilot, and Perplexity can respond to natural-language prompts, summarize clinical information, draft documents and educational materials, interpret clinical scenarios, and propose preliminary treatment plans [1,2]. These capabilities make LLMs attractive as flexible decision-support tools, but the same free-text generation process can produce hallucinations, loss of context, inaccurate citations, and unwarranted confidence [3,4]. Research on hallucination mitigation and studies of fabricated medical references further show that fluent output is not equivalent to reliable evidence [5,6].

This distinction is especially important in rehabilitation. Rehabilitation decisions integrate impairment, activity limitations, participation restrictions, environmental barriers, patient goals, recovery stage, pain response, comorbidity, and functional adaptation over time. A response may therefore appear clinically plausible yet be incomplete, insufficiently individualized, or unsafe for a particular patient. Evaluation in this field must extend beyond simple answer accuracy to include clinical relevance, completeness, applicability, guideline concordance, individualization, safety, and the extent of expert correction required before use.

Recent reviews have begun to examine LLMs and generative AI in rehabilitation, but they have generally focused on specific models, functions, or narrower segments of the rehabilitation process [7,8]. As a result, the field still lacks a comprehensive, rehabilitation-specific map that shows where empirical evidence is concentrated, how models and prompts are reported, which outcomes are used, and whether the available findings support educational, clinician-facing, or patient-facing applications. General medical benchmarks cannot be assumed to transfer directly to rehabilitation tasks such as exercise prescription, ICF coding, functional goal-setting, or adaptive treatment planning.

The broader medical AI literature indicates that clinical usefulness depends not only on accuracy but also on safety, transparency, explainability, workflow integration, and human oversight [9,10,11]. For rehabilitation, the central question is therefore not simply which model achieves the highest score, but which tasks are sufficiently bounded and reviewable, which methodological conditions support trustworthy performance, and where current evidence is insufficient to support clinical implementation. A rehabilitation-specific synthesis is needed to distinguish promising assistive functions from applications that still require substantial validation and safeguards.

Accordingly, this review limited the evidence base to peer-reviewed, full-text original empirical studies and systematically mapped applications of generative AI and LLMs across rehabilitation assessment, clinical reasoning, decision support, planning, education, functional classification, and professional support. It also examined reporting of models and versions, prompting and implementation strategies, reproducibility variables, outcome measures, safety-related findings, methodological limitations, funding, and the extent of expert oversight.

The specific objectives were to characterize the general and methodological features of the included studies; summarize the AI models and prompting or implementation strategies; integrate the major rehabilitation application and outcome domains; and critically identify the current contributions, limitations, implementation boundaries, and future research needs for rehabilitation practice and education.

This review aimed to answer the following research questions:

(1) What are the general study characteristics and methodological quality of research on generative AI and LLMs in rehabilitation?
(2) What AI models and prompting or implementation strategies were used in the included studies?
(3) In which rehabilitation contexts and major application domains have generative AI and LLMs been evaluated?
(4) Which outcome domains and measures were used in the included studies?
(5) What possibilities, limitations, and implications does the current evidence offer for clinical rehabilitation practice, education, and future research?

## 2. Materials and Methods

### 2.1. Study Design

This scoping review was conducted in accordance with the Joanna Briggs Institute (JBI) scoping review methodology [12] and reported using the PRISMA Extension for Scoping Reviews (PRISMA-ScR) checklist [13]. The JBI methodology guided the selection of the scoping review design, the establishment of eligibility criteria based on the PCC framework, standardized data charting, narrative synthesis, mapping of application domains, and identification of research gaps. The purpose of this study was not to pool effect sizes or treatment effects for a specific intervention, but to systematically map how generative AI and large language models have been applied and evaluated across rehabilitation-related clinical, educational, assessment, planning, functional-classification, and decision-support contexts. A scoping review design was therefore chosen to explore this emerging and heterogeneous evidence base, characterized by diverse study designs, populations, AI models, prompting strategies, comparators, and outcomes. Eligibility criteria were predefined for this review using the Population–Concept–Context (PCC) framework in accordance with JBI guidance. This review was registered on the Open Science Framework (OSF) using the Open-Ended Registration format (https://doi.org/10.17605/OSF.IO/2J3ZB; registration date: 9 July 2026). Because this registration was completed after the initial bibliographic database search, it should be interpreted not as prospective protocol registration but as a methodological record intended to increase the transparency of the search, screening, data extraction, and synthesis procedures.

### 2.2. Eligibility Criteria for Study Inclusion

Eligibility criteria for this review were predefined to align with the study purpose and were structured using the Population–Concept–Context (PCC) framework. Eligible studies evaluated generative AI or LLM-based systems for rehabilitation-related assessment, clinical reasoning, decision support, rehabilitation planning, education, simulation, technical validation, or other rehabilitation-specific professional applications. Peer-reviewed, full-text original empirical studies were eligible, whereas studies without a generative AI/LLM component and non-primary or non-peer-reviewed evidence were excluded. Detailed inclusion and exclusion criteria for population, concept, context, evidence type, language, and publication period are presented in Table 1. The search was restricted to English-language literature published between 1 January 2015 and 24 July 2026.

### 2.3. Search Strategy

The search was conducted through 24 July 2026 and covered English-language literature published between 1 January 2015 and 24 July 2026. The databases searched were PubMed/MEDLINE, CINAHL, Web of Science Core Collection, Scopus, and IEEE Xplore. The search strategy comprised two core concept blocks: generative AI/large language models and rehabilitation/functional and rehabilitation context. In PubMed/MEDLINE, MeSH terms were combined with title/abstract ([tiab]) terms; terms within each concept were combined with OR, and the two concept blocks were combined with AND. Specifically, the final PubMed/MEDLINE search was constructed by combining the generative AI/LLM block with the rehabilitation/functional and rehabilitation context block, with limits applied for English language and publication date from 1 January 2015 to 24 July 2026. In CINAHL, Web of Science Core Collection, Scopus, and IEEE Xplore, the same core concepts were retained, with search syntax adjusted to each database’s indexing system and search fields. The PubMed/MEDLINE search strategy is presented in Table 2, and the complete executable search strategies for all databases are provided in Supplementary Table S1.

### 2.4. Study Selection

Study selection was conducted independently by two reviewers: the primary researcher and an external rehabilitation reviewer. The external reviewer was a licensed occupational therapist with a doctoral degree and more than 20 years of clinical and research experience in rehabilitation, with no financial or academic conflicts of interest related to the included studies or the review process. After duplicate removal, each reviewer independently screened all titles and abstracts using the predefined PCC and evidence-type criteria. Potentially eligible reports were then retrieved in full and independently assessed by both reviewers using the same criteria. Initial decisions were compared only after each reviewer completed their independent assessment; disagreements were resolved by re-examining the source and reaching consensus, and one principal reason for full-text exclusion was recorded. Inter-rater agreement for screening and full-text eligibility was calculated from the two reviewers’ pre-consensus judgments.

### 2.5. Data Extraction Process

Data extraction was independently performed by the primary researcher and one external rehabilitation reviewer using the same standardized Microsoft Excel 365 form. The form was piloted on three likely eligible studies and refined before full extraction. Each reviewer independently extracted author, publication year, country, publication type, study design, rehabilitation field, participant/case characteristics, AI model and version, interface/deployment, access date, prompting or implementation strategy, repeated-run procedures, generation settings, evaluation instrument, key findings, safety/clinical limitations, and funding. The two independently completed extraction records were then compared, and discrepancies were resolved by re-checking the source article and reaching consensus. All extraction judgments were made by the two reviewers.

In this review, “AI model” refers to the name and version of the generative AI, LLM, or LLM-based system specified in each included study; when the model version was not clearly reported, the name as given in the original text was recorded verbatim. “Prompting or implementation strategy” was defined as the procedure used to generate or implement the AI response, including clinical questions, guideline-based questions, hypothetical cases, standardized scenarios, zero-shot, few-shot, chain-of-thought, role-play, retrieval-augmented generation, or conversion of a clinician’s prescription into software logic. “Rehabilitation context” was defined as clinical or educational settings related to rehabilitation, including physical therapy, occupational therapy, speech-language pathology, physical medicine and rehabilitation, cardiac rehabilitation, vestibular rehabilitation, musculoskeletal rehabilitation, neurorehabilitation, and ICF-based functional classification. “Outcome” referred to the primary outcome measures reported in the included studies, such as accuracy, guideline adherence, quality of clinical reasoning, appropriateness of treatment plans, safety, consistency, reliability, usability, educational effectiveness, and technical classification performance. Multiple publications that appeared to arise from the same underlying participant cohort were linked as companion reports. Report-level data extraction and methodological appraisal were retained separately when companion publications addressed distinct analyses. Publication year, study design, methodological appraisal, reporting completeness, and funding were summarized at the publication level (*n* = 43), whereas rehabilitation context, model family, application domain, and outcome domain counts were summarized at the unique-study level (*n* = 42). For non-mutually exclusive categories, each unique study was counted at most once per category.

Each study was assigned to a mutually exclusive primary application domain using a hierarchy based on its principal objective, primary outcome, and intended rehabilitation user or context. Information not reported or not confirmed in a source was recorded as “not reported.” When study design or publication type was unclear, classification was determined by reviewer consensus based on the methods, population, comparative structure, and evaluation approach described in the original text.

### 2.6. Study Quality Assessment

For quantitative, descriptive, and mixed-methods studies, the Mixed Methods Appraisal Tool (MMAT, 2018) was used, with criteria specific to each study design [14]. Randomized controlled trials were assessed with the revised JBI critical appraisal tool for randomized controlled trials [15]. Quasi-experimental studies were assessed with the revised JBI critical appraisal tool for quasi-experimental studies [16]. Case reports were evaluated with the JBI Critical Appraisal Checklist for Case Reports [17]. The diagnostic/classification validation study was assessed with QUADAS-3, which evaluates risk of bias and applicability in diagnostic test accuracy studies [18].

The primary researcher and the external rehabilitation reviewer independently completed all design-specific appraisal items before comparing their judgments. Inter-rater agreement for methodological appraisal was calculated as percentage agreement across applicable item-level judgments, based on the two reviewers’ independent pre-consensus assessments. Disagreements were resolved through discussion and consensus, and the two reviewers made all appraisal judgments. Appraisal judgments were interpreted at the individual item or domain level rather than by calculating an overall numerical quality score. No arbitrary high-, moderate-, or low-quality cut-offs were applied, and studies were not excluded solely on the basis of methodological appraisal. Instead, appraisal findings were used to characterize methodological strengths and limitations, as well as the certainty with which individual study findings could be interpreted. Detailed study-level appraisal results are presented in Supplementary Table S2.

### 2.7. Synthesis of Results

Because the design, populations, AI models, prompting strategies, comparators, and outcomes of the included studies were heterogeneous, a meta-analysis was not conducted. Instead, the findings were synthesized narratively in accordance with the JBI scoping review methodology, and the PCC framework was used to map rehabilitation contexts, AI applications, and outcomes [12].

Data were charted using predefined items, including study characteristics, rehabilitation context, AI model and implementation strategy, application purpose, comparator, and outcome measures. For the application-domain synthesis, initial codes were derived inductively from each study’s stated objective, AI/LLM task, and principal outcome, rather than from keyword frequency alone. These codes were iteratively compared and consolidated into six mutually exclusive primary application domains. A coding framework was developed, specifying the initial/working codes, operational definitions, primary-domain assignment rules, ambiguity-resolution rules, and representative studies.

Each study was assigned a single primary application domain using a predefined hierarchy: (1) principal study objective, (2) principal evaluated outcome, and (3) intended rehabilitation user or context. Secondary functions were recorded separately and excluded from the primary-domain count. The primary researcher and an external rehabilitation reviewer independently coded each included study, then compared their assignments. Potentially overlapping cases were re-examined in full and resolved through discussion and consensus, guided by the operational definitions. Inter-coder agreement for the six-domain classification was assessed using percentage agreement and Cohen’s κ, calculated exclusively from the two reviewers’ pre-consensus classifications. Descriptive frequencies were calculated at the publication level for publication characteristics and reporting variables, and at the unique-study level for rehabilitation context, model family, application domain, and outcome domain. Companion publications were consolidated before calculating unique-study frequencies.

## 3. Results

### 3.1. Literature Search and Selection

The updated database search identified 2126 records: 1177 from Scopus, 525 from PubMed/MEDLINE, 220 from Web of Science Core Collection, 149 from CINAHL/EBSCO, and 55 from IEEE Xplore. After removing 600 duplicate records, 1526 records were screened by title and abstract. Of these, 1391 were excluded per the predefined eligibility criteria, leaving 135 full-text reports for eligibility assessment.

**Figure 1:**
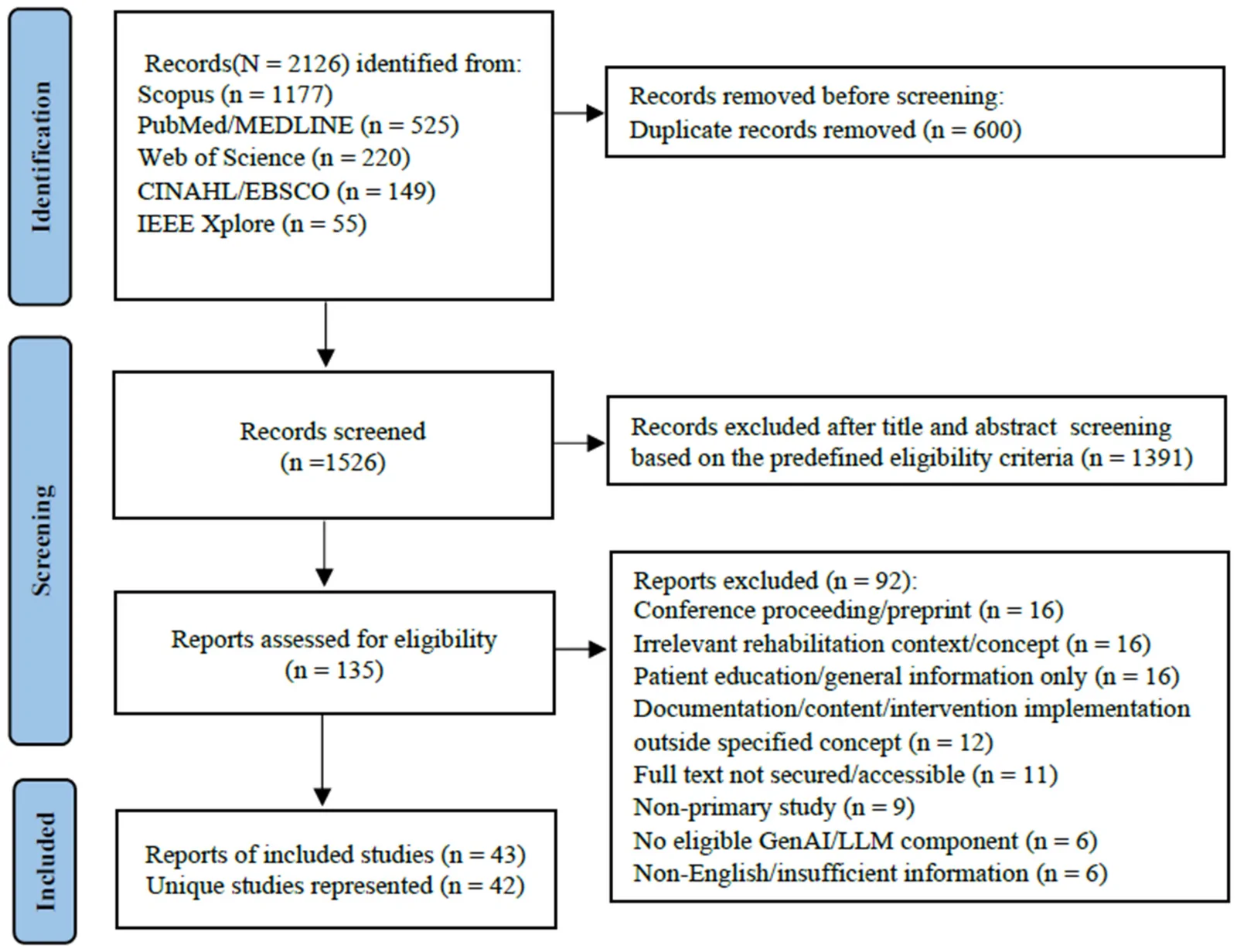
PRISMA flow diagram illustrating the study identification, screening, eligibility, and inclusion process.

After full-text review, 92 reports were excluded for the following reasons: conference proceedings or preprints (*n* = 16), irrelevant rehabilitation context or concept (*n* = 16), patient education or general information only (*n* = 16), documentation, content generation, or intervention implementation outside the specified concept (*n* = 12), full text not secured or accessible (*n* = 11), non-primary study (*n* = 9), no eligible generative AI or LLM component (*n* = 6), and non-English language or insufficient information (*n* = 6). A total of 43 publications representing 42 unique studies were included in this scoping review (Figure 1).

Title/abstract screening and full-text eligibility assessment were conducted independently by one primary researcher and one external rehabilitation reviewer, using the prespecified PCC and evidence-type criteria. Initial judgments were made separately by the two reviewers, and disagreements were resolved by discussion and consensus after each stage. At title/abstract screening, the reviewers agreed on 1448 of 1526 records and disagreed on 78, yielding 94.89% agreement and Cohen’s κ = 0.726. At full-text eligibility assessment, 123 of 135 reports were concordantly classified, and 12 were discordant, yielding 91.11% agreement and Cohen’s κ = 0.800. A single principal reason for exclusion was assigned after consensus at the full-text stage.

### 3.2. Methodological Appraisal Results

Design-specific appraisal showed that most included studies were quantitative, descriptive model-output or benchmarking studies assessed with MMAT (*n* = 31), followed by mixed-methods studies (*n* = 5), JBI quasi-experimental studies (*n* = 2), JBI randomized controlled trials (*n* = 2), JBI case reports (*n* = 2), and one QUADAS-3 diagnostic/classification validation study (Table 3). Across all applicable methodological-appraisal items, the pre-consensus percentage agreement between the two reviewers was 88.9%. All disagreements were subsequently resolved through discussion and re-examination of the original studies. Across descriptive evidence, recurrent concerns included purposive or narrow case/question sets, study-specific rating frameworks, and limited external validation. Educational trials offered stronger comparator structures but were constrained by infeasible blinding, attrition, or temporal/cohort effects. Appraisal was interpreted at the item/domain level without arbitrary summed quality thresholds, and no study was excluded solely because of appraisal findings. Detailed study-level judgments and source-based explanations are provided in Supplementary Table S2.

### 3.3. General Characteristics of Studies

The evidence base was very recent: 17 studies were published in 2026, 21 in 2025, 4 in 2024, and 1 in 2023. These 43 publications [19,20,21,22,23,24,25,26,27,28,29,30,31,32,33,34,35,36,37,38,39,40,41,42,43,44,45,46,47,48,49,50,51,52,53,54,55,56,57,58,59,60,61] represented 42 unique studies because the two Gürses et al. [43,44] reports used a shared knee osteoarthritis cohort (Supplementary Table S3). The evidence covered physical therapy, occupational therapy, physical medicine and rehabilitation, speech-language pathology, musculoskeletal and sports rehabilitation, vestibular and cardiac rehabilitation, neurorehabilitation, renal rehabilitation, and rehabilitation education. Most studies evaluated structured model outputs, clinical scenarios, guideline concordance, expert ratings, or educational outcomes rather than real-world patient care.

**Figure 2:**
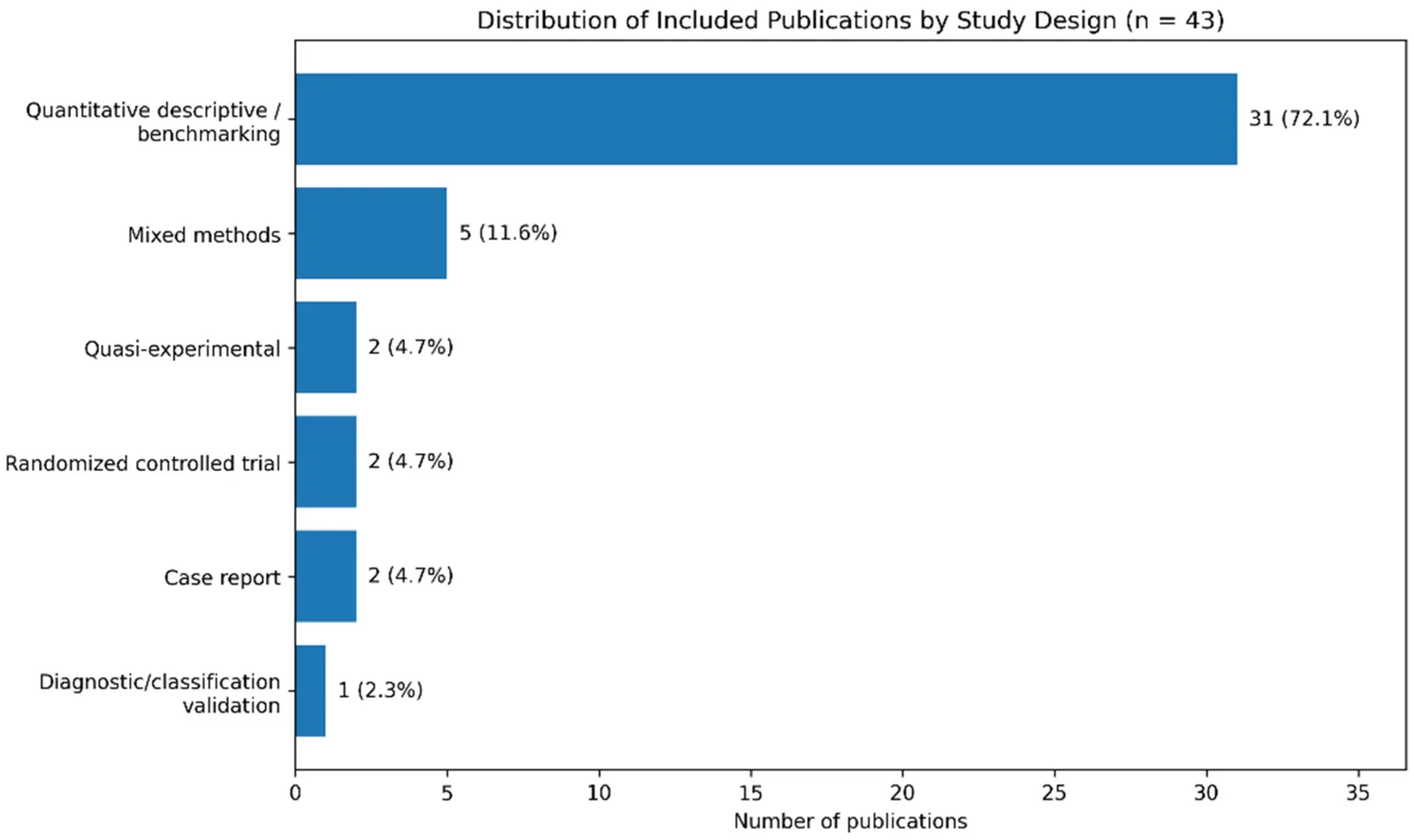
Distribution of included publications by study design.

**Figure 3:**
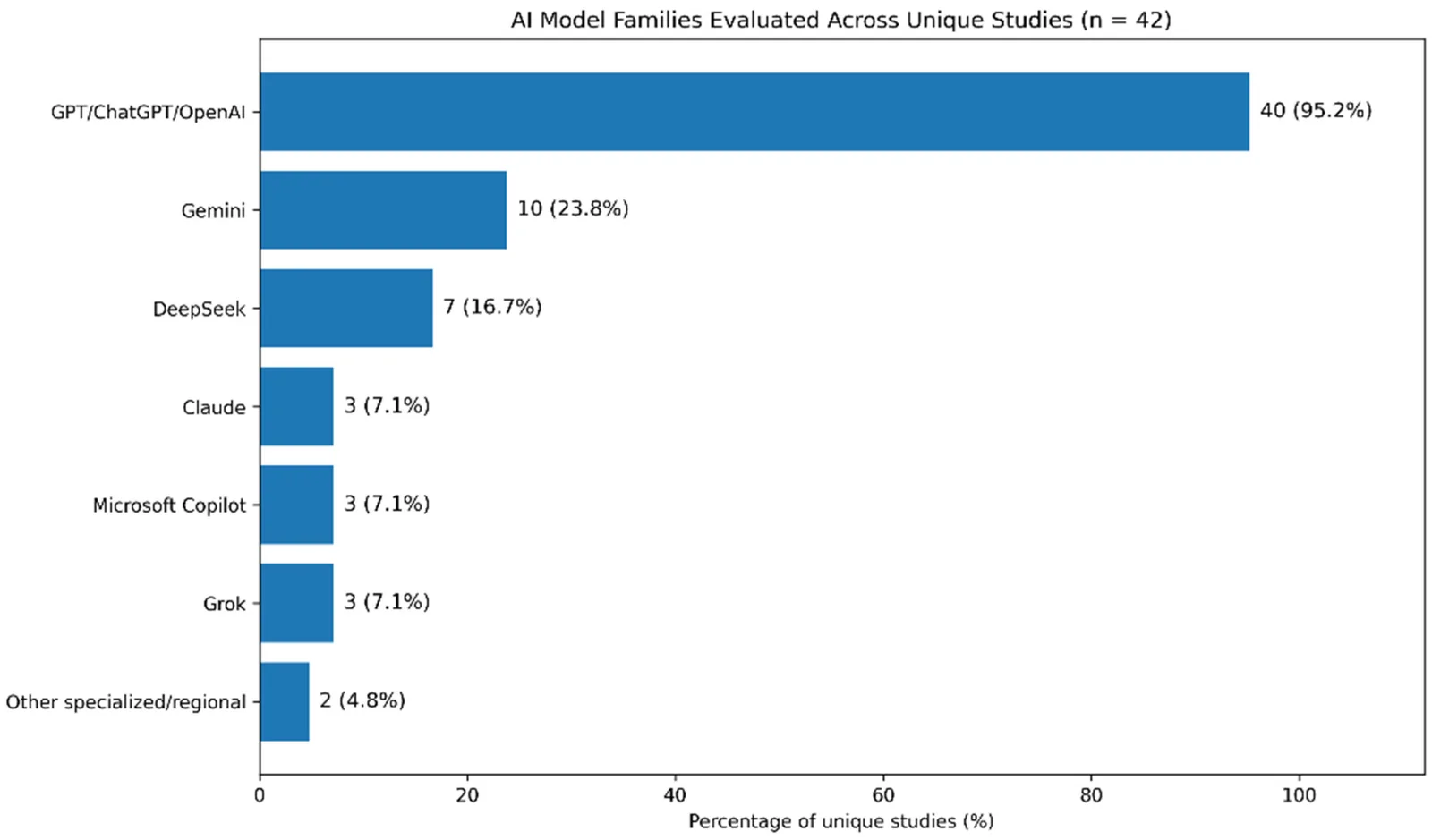
AI model families evaluated across the unique studies.

**Figure 4:**
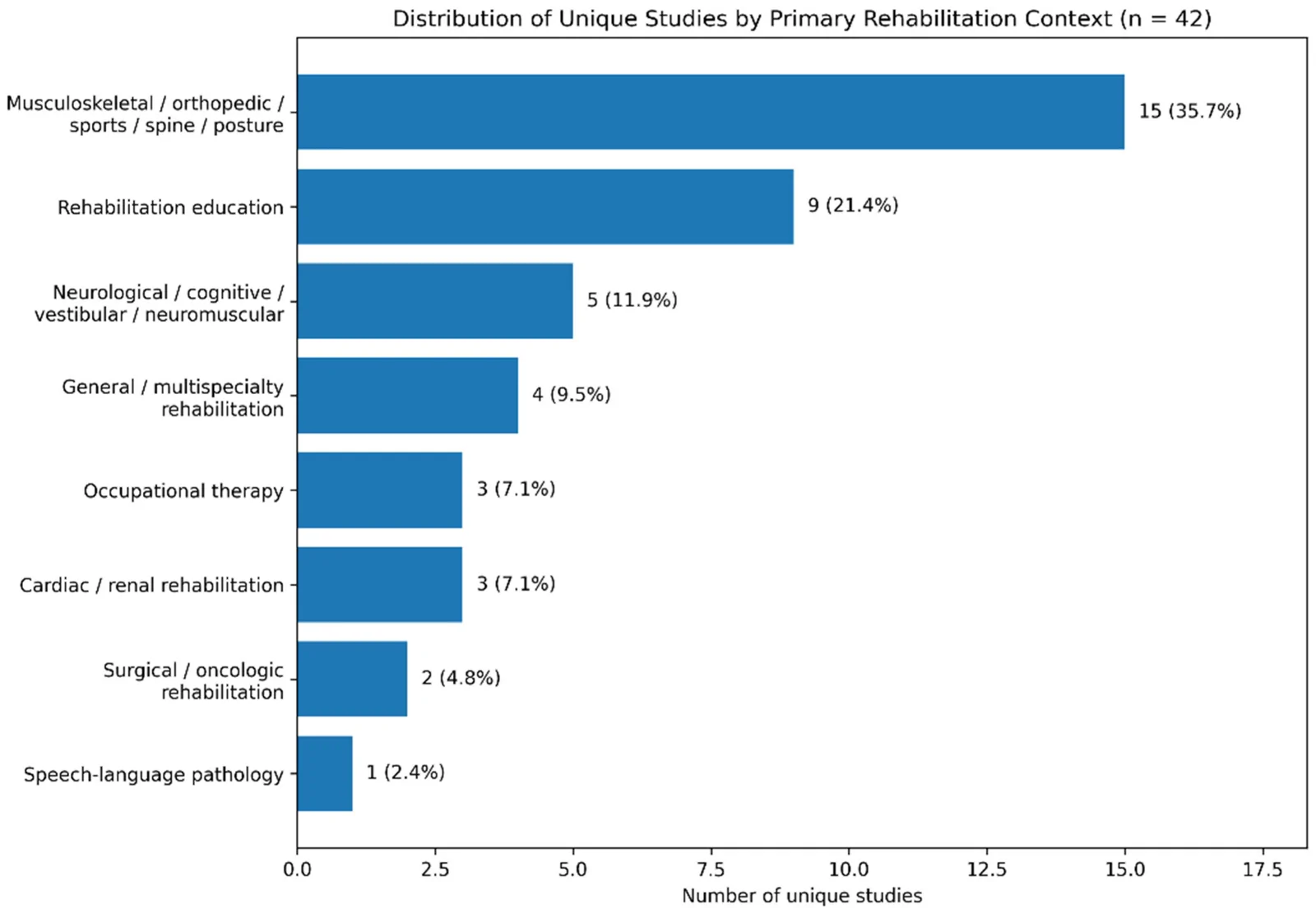
Distribution of unique studies by primary rehabilitation context.

Study design was dominated by quantitative descriptive and benchmarking publications (31/43, 72.1%), followed by mixed-methods studies (5/43, 11.6%). Quasi-experimental studies, randomized controlled trials, and case reports each accounted for two publications (4.7%), and one publication (2.3%) was a diagnostic/classification validation study (Figure 2). At the unique-study level, GPT/ChatGPT/OpenAI-family systems were evaluated in 40 of 42 unique studies (95.2%), followed by Gemini in 10 (23.8%) and DeepSeek in seven (16.7%); model-family counts were not mutually exclusive (Figure 3). At the unique-study level, the largest primary context was musculoskeletal, orthopedic, sports, spine, and postural rehabilitation (15/42, 35.7%), followed by rehabilitation education (9/42, 21.4%) and neurological, cognitive, vestibular, or neuromuscular rehabilitation (5/42, 11.9%) (Figure 4). Detailed information on model version, access, prompt, repeated runs, generation settings, and evaluation is provided in Supplementary Table S4. The major study objectives, key results, and main findings are presented in Supplementary Table S5.

### 3.4. Mapping of Application Domains of Generative AI and LLMs in Rehabilitation

Application domains were derived from each study’s stated objective, AI/LLM task, and principal outcome. Initial codes were generated inductively and iteratively, then compared and consolidated into six operational domains. To improve classification consistency, a coding framework was developed that specified initial/working codes, operational definitions, primary assignment rules, and tie-break rules for ambiguous cases (Supplementary Table S6).

Each publication was initially assigned a single, mutually exclusive primary application domain based on the hierarchy of principal objective, principal evaluated outcome, and intended rehabilitation user/context. The primary researcher and an external rehabilitation reviewer independently coded all 43 publications and then compared their assignments. The two reviewers agreed on the primary-domain classification for 38 of 43 publications (88.37%), with five initial disagreements, yielding a Cohen’s κ = 0.850. Ambiguous classifications were resolved through full-text re-examination and consensus. Companion publications derived from the same underlying cohort were linked and counted only once in study-level domain totals. Consequently, the 43 included publications represented 42 unique studies for application-domain mapping.

Using this coding framework, the 43 included publications were consolidated into 42 unique studies for the application-domain synthesis (Table 4). At the unique-study level, clinical reasoning and decision support was the most frequent domain (*n* = 13), followed by rehabilitation education, simulation, and feedback (*n* = 10), rehabilitation planning and prescription (*n* = 9), guideline adherence and clinical-question support (*n* = 6), adaptive feedback and rehabilitation support (*n* = 2), and assessment and functional classification (*n* = 2). These mutually exclusive unique-study counts summed to 42 because the two companion Gürses publications were consolidated into a single study within the rehabilitation planning and prescription domain. Detailed participant characteristics, model information, evaluated tasks, and study-level findings are provided in Supplementary Tables S3–S5.

### 3.5. AI Models and Prompt or Implementation Strategies

At the unique-study level, GPT/ChatGPT/OpenAI-family systems were evaluated in 40 of 42 unique studies (95.2%), followed by Gemini in 10 (23.8%) and DeepSeek in seven (16.7%). Claude, Microsoft Copilot, and Grok were each evaluated in three unique studies (7.1%), and other specialized or regional systems in two (4.8%) (Table 5). Because multiple model families could be evaluated within a single study, the model-family counts were not mutually exclusive. Prompting ranged from direct questions and structured clinical vignettes to guideline-derived, role-based, sequential SOAP, zero-shot/few-shot, multilingual, multimodal, and agent-supported strategies. Product names, interfaces, subscription labels, and actual model versions were separated wherever possible, and unreported model snapshots or generation parameters were not inferred. Detailed information on model versions, prompting strategies, interfaces, and reported generation settings for each publication is provided in Supplementary Table S4.

### 3.6. Outcome Domains and Measures

Outcome domains overlapped across the 42 unique studies. Companion publications from the same underlying cohort were consolidated, and each unique study was counted at most once per outcome domain. The most common domain was accuracy, guideline adherence, or agreement with an expert or reference standard (*n* = 33), followed by clinical reasoning, decision quality, treatment planning, or prescription quality (*n* = 25). Direct patient-level outcome evidence remained extremely limited (Table 6).

### 3.7. Model Reporting, Reproducibility, Safety, and Evidence Gaps

**Figure 5:**
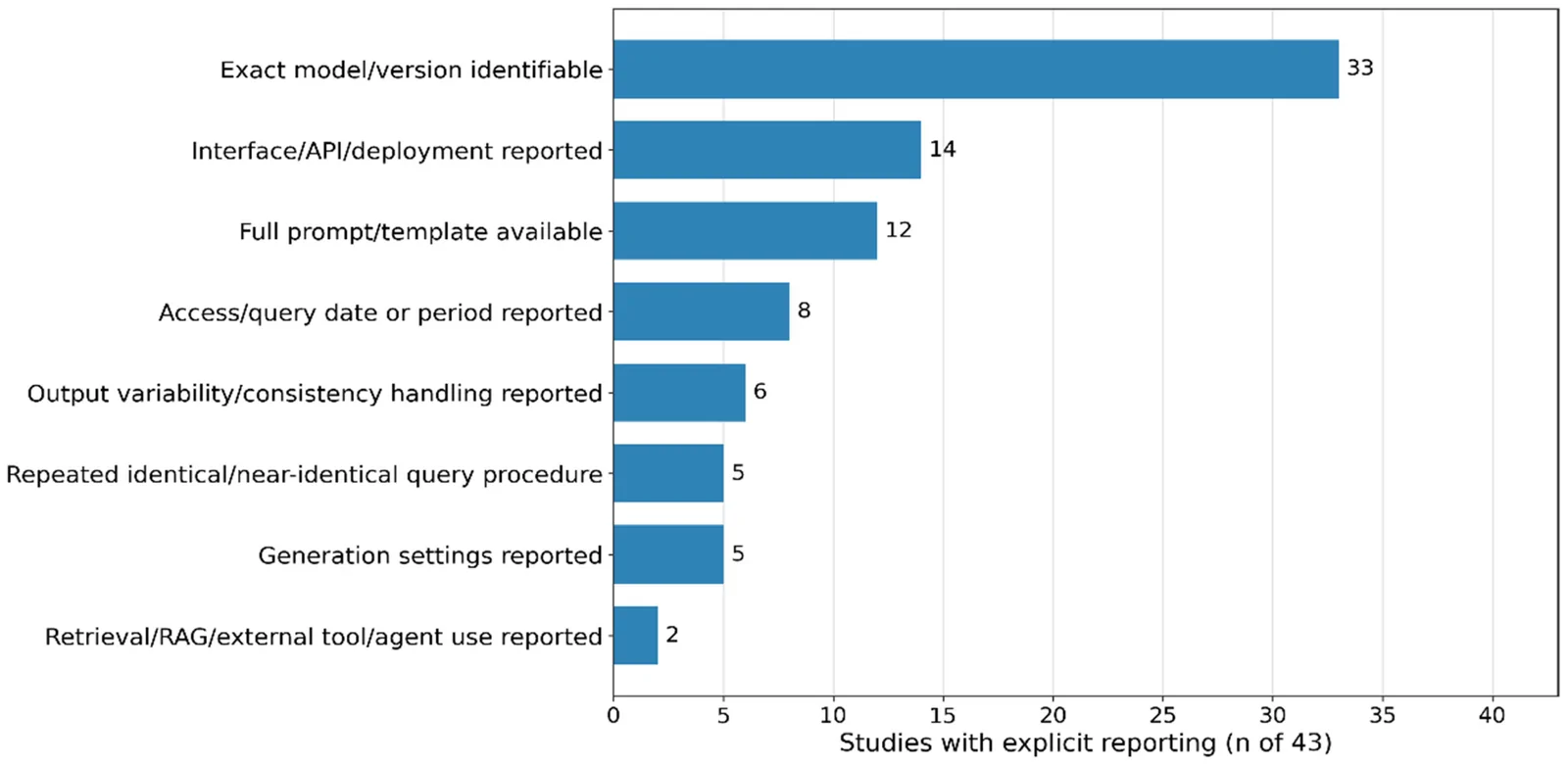
Reporting completeness for model- and prompting-related reproducibility variables.

Reporting completeness varied substantially across the 43 studies. An exact model or version was identified in 33 studies, and interface/API/deployment information in 14; only 12 provided a full prompt or template, eight reported an access/query date or period, six described how output variability or consistency was handled, five reported repeated identical or near-identical query procedures, five reported generation settings, and two explicitly described retrieval/RAG/external-tool or agent use. These gaps limit temporal reproducibility and direct cross-study comparison because model outputs may vary with model snapshot, interface, access date, prompting, and generation conditions. NR was used whenever the source did not explicitly report a variable (Figure 5, Supplementary Table S4). Funding information was reported in 40 of the 43 studies. Eleven studies reported research, project, or investigator support, including two that also reported open-access publication support; 25 explicitly reported no research or external financial support; and four reported publication or open-access support only. Funding information was not reported in the remaining three studies, which were coded as NR (Figure 5, Supplementary Table S4). Safety-focused evaluation was also incomplete. Red-flag detection, contraindication recognition, inappropriate exercise progression, referral/escalation logic, citation reliability, and hallucination were not systematically assessed across studies (Supplementary Table S5).

## 4. Discussion

The central finding of this review is a task-readiness gradient rather than uniform clinical readiness. Generative AI and LLMs performed most consistently on bounded, reviewable tasks with explicit reference standards, such as guideline-derived questions, predefined educational exercises, and structured classification tasks [25,42,52,54,59]. Their performance was less reliable for tasks requiring individualized rehabilitation planning, longitudinal adaptation, integration of comorbidities, or safety-sensitive clinical judgment [20,23,24,43,49,51]. Accordingly, the current evidence supports task-specific assistive use rather than broad or autonomous clinical deployment. Adjacent reviews likewise indicate that benchmark and proxy outcomes substantially outnumber prospective clinical evaluations [62,63,64,65].

Prompting is integral to the intervention and a major source of both performance variation and reproducibility failures. Changes to the query language affect program structure and guideline alignment [20], structured remediation improves selected dimensions of low-back-pain reasoning [30], and few-shot examples alter the perceived usefulness of educational feedback [54]. Prompt-pattern research also shows that output behavior can be deliberately shaped by prompt structure [66]. However, incomplete reporting of prompts, model snapshots, access dates, repeated runs, and generation settings makes it difficult to determine whether an apparent result reflects the model, the prompt, the interface, or a transient software version. Rehabilitation studies should therefore treat prompt design, version control, and handling of output variability as core methodological variables rather than supplementary technical details [66,67].

### 4.1. Task-Dependent Performance and Methodological Interpretation

No model demonstrated consistent superiority across rehabilitation tasks. Newer GPT versions outperformed older ones in some comparisons [34,53]; DeepSeek performed better in selected postoperative cases [22]; and Grok-4, Gemini-2.5-Pro, or regional models received high ratings in specific benchmarks [21,29]. These findings are better interpreted as context-specific advantages rather than a durable hierarchy of clinical capability. Language, prompt optimization, task format, evaluator expectations, model recency, and access conditions were rarely isolated, so the source of apparent superiority remains uncertain. Model selection should therefore be based on validated performance for the intended rehabilitation task and population, not on a single leaderboard-style comparison.

Methodological design often yields overly optimistic or unstable estimates. Clear or typical cases can overstate generalizability; small question sets produce fragile rankings; author-developed rubrics may reward the response style investigators anticipate; non-blinded evaluation can introduce expectation bias; and single-run testing ignores stochastic variation in output. Mean scores are also clinically insufficient because they can conceal rare but consequential failures, such as omitted red flags, unsafe progression, contraindicated recommendations, and fabricated references.

The independence of the evidence base also requires careful interpretation. Multiple publications from a single cohort can create an impression of replication, even though they represent different analyses of the same underlying data. Consolidating companion reports at the unique-study level reduced this inflation in the present review, but unidentified overlap may remain. Future syntheses should clearly distinguish among publications, datasets, model-output sets, and independent clinical cohorts to avoid overstating the quantity and consistency of evidence.

### 4.2. Clinical Validity and Limitations of Applicability to Practice

The principal translational limitation is the gap between performance on structured benchmarks and performance in routine rehabilitation care. Most included studies used static or simulated cases, curated datasets, or guideline-derived questions, and assessed accuracy, agreement, or expert-rated content quality. These designs can demonstrate technical feasibility, but they do not replicate longitudinal rehabilitation practice, in which decisions must be revised in response to pain, fatigue, cognition, comorbidity, caregiver capacity, adherence, environmental barriers, and changes in medical status.

Clinical applicability was further constrained by limited external validation, inconsistent guideline concordance, unreliable references, incomplete testing of red flags and contraindications, and insufficient personalization of dosage, progression, and feasibility [20,23,24,25,43,49,51,52,59]. Only one included study directly assessed a patient-level outcome, and evidence was largely lacking for function, participation, quality of life, adverse events, adherence, workflow efficiency, cost, equity, and the therapeutic relationship. Therefore, favorable model-output evaluations should be interpreted as preliminary evidence of task performance, not as evidence of clinical effectiveness or readiness for autonomous use.

### 4.3. Risk-Stratified Rehabilitation and Digital Implementation

The most defensible implementation strategy is risk-stratified, clinician-supervised integration rather than unrestricted deployment. Generative AI may add value by reducing the burden of information processing or drafting while keeping clinical authority with the rehabilitation professional. Its usefulness should be judged by workflow fit, clinician workload, patient access, digital literacy, privacy, equity, interoperability, and whether the tool improves decisions or outcomes without weakening the therapeutic relationship or professional accountability [68]. The risk categories below are the author’s interpretation of the reviewed evidence and are intended to clarify implementation boundaries rather than serve as a formal regulatory classification.

Lower-risk applications are those in which the output is educational, administrative, or readily reviewable before it affects care. Examples include drafting case materials, generating formative feedback, organizing documentation, and summarizing guideline content. Structured educational studies have reported improvements in knowledge, engagement, perceived usefulness of feedback, and AI self-efficacy [31,35,40,41,47,50,54,58]. Even in these settings, instructor or clinician verification remains necessary to prevent subtle factual errors, over-reliance, and the erosion of independent reasoning.

Moderate-risk applications include clinician-facing assessment support, functional classification, and draft rehabilitation plans. Evidence from occupational therapy, knee osteoarthritis, postoperative rehabilitation, cardiac rehabilitation, and classification tasks indicates that LLMs can organize options and generate plausible starting points, but expert agreement and clinical specificity remain limited [22,24,42,43,49,61]. These tools are best used as second readers or drafting aids, with professionals verifying evidence, individualizing goals and dosing, and documenting the rationale for the final decision.

Higher-risk applications include autonomous triage, red-flag interpretation, contraindication screening, exercise dosing and progression, return-to-activity decisions, and direct patient-facing advice that can alter treatment without review. Real-patient triage findings and guideline comparisons indicate that fluent responses may systematically diverge from professional decisions or evidence-based recommendations [25,51,52,56]. These functions require validated safety endpoints, explicit escalation rules, clinician override, prospective monitoring, and a demonstrated ability to refrain when information is insufficient.

Overall, the evidence supports clinician-supervised assistance rather than autonomous decision-making. Rehabilitation professionals should retain final authority over interpretation, prescribing, progression, and safety-critical decisions. The proposed risk staging is a conceptual synthesis of the reviewed evidence and should not be construed as a prospectively validated or formal medical-device risk classification [11,69,70,71].

### 4.4. Safety, Governance, and Operational Human Oversight

Average accuracy and mean expert ratings are inadequate safety indicators. A model can achieve a favorable overall score while generating an infrequent contraindicated recommendation, fabricating a citation, or omitting a red flag. Guideline mismatch, incomplete rehabilitation specificity, expert modification requirements, and reference inaccuracy were directly observed in the included evidence [25,43,49,51,52,59]. Safety evaluation should therefore emphasize failure modes and consequences through worst-case analysis, contraindication and escalation tests, citation verification, assessment of uncertainty or abstention behavior, repeated-run consistency, and documentation of whether a human reviewer detected the error before patient impact [72].

Governance must translate into operational controls. Principles of autonomy, transparency, accountability, inclusiveness, responsiveness, sustainability, and protection of human well-being [73], together with guidance for contemporary generative systems and trustworthy healthcare AI [69,71], imply concrete requirements for model and version logging, traceable source use, role-based access, privacy protection, clinician sign-off, explicit escalation pathways, monitoring of overrides and near misses, and periodic revalidation after updates. Explainability is also relevant because a system may remain unsuitable for high-stakes use if clinicians cannot understand or challenge the basis of its recommendations [74].

Human oversight should be documented as a reproducible operational process, including reviewer qualifications, review timing, mandatory verification items, override criteria, disagreement procedures, and responsibility for the final decision. These details are necessary to determine whether human-in-the-loop arrangements meaningfully reduce automation bias and patient risk.

### 4.5. Limitations

Several limitations should be considered. First, the updated search used five complementary databases—PubMed/MEDLINE, CINAHL/EBSCO, Web of Science Core Collection, Scopus, and IEEE Xplore—to cover biomedical, allied health, multidisciplinary, and engineering literature. However, additional sources such as Embase, ACM Digital Library, and PsycINFO were not searched, and relevant studies indexed primarily in those databases may have been missed. Restricting the search to English-language, peer-reviewed, full-text original empirical studies improved consistency in evidentiary status but may have introduced language bias and reduced sensitivity to rapidly emerging technical work reported only as conference papers or preprints.

Second, formal publication-bias testing was inappropriate because comparable effect estimates were not pooled. Nevertheless, publication and selective-reporting biases remain plausible. Favorable model performance may be more likely to be submitted or published, whereas failed prompts, unsafe outputs, null comparisons, and unfavorable repeated runs may be omitted or incompletely reported. The peer-reviewed-only criterion may therefore have increased confidence in source status while simultaneously favoring positive findings.

Third, the evidence was heterogeneous and predominantly based on model outputs, simulated cases, and comparisons with experts or guidelines. Differences in models, prompts, access dates, reference standards, languages, evaluators, and outcome measures precluded quantitative synthesis and limited interpretation of frequencies or apparent model advantages. Although known companion publications were consolidated at the unique-study level, overlapping datasets or output sets that were not recognized cannot be fully excluded.

Fourth, the evidence’s applicability to routine clinical practice was limited. Most studies evaluated single-turn or short-horizon outputs under controlled conditions, relied on surrogate outcomes, and did not assess integration with electronic health records, privacy, interoperability, professional liability, clinician workload, equity, or the therapeutic relationship. Consequently, even studies reporting high accuracy or favorable expert ratings cannot establish that an LLM will improve rehabilitation outcomes or remain safe in evolving real-world clinical conditions.

### 4.6. Future Research Directions

A staged research roadmap is needed to advance from promising demonstrations to clinically credible implementation. Stage 1 should establish reproducible task benchmarks using prespecified prompts, expert reference standards, repeated runs, failure-mode analysis, and explicit safety endpoints. Stage 2 should conduct prospective silent-mode validation using real rehabilitation data without influencing care. Stage 3 should evaluate supervised human–AI workflows, including decision quality, time, usability, clinician workload, overrides, near misses, equity, and cost. Stage 4 should advance to multicenter prospective or pragmatic trials measuring patient-centered function, participation, adverse events, adherence, workflow effects, and long-term outcomes. Stage 5 should address regulatory lifecycle management and post-deployment surveillance through drift monitoring, incident reporting, audit logs, explainability review, and revalidation after model or interface updates.

Across all stages, studies should report the exact model and version, access method and date, full prompts and system instructions, generation settings, repeated-run procedures, external tools, and the operational human-review process, in line with emerging LLM reporting guidance. Rehabilitation-specific reporting should also describe patient goals, functional context, contraindications, escalation criteria, and whether AI output changed the clinician’s final decision. Research should extend beyond predominantly musculoskeletal and physical-therapy settings and prioritize comparisons of AI-assisted workflows with usual care. Outcomes should include patient function, participation, adverse events, adherence, clinician workload, cost, equity, and long-term safety.

## 5. Conclusions

This scoping review identified 43 peer-reviewed empirical publications representing 42 unique studies on generative AI and LLMs across rehabilitation assessment, clinical reasoning and decision support, rehabilitation planning, education, guideline support, adaptive feedback, and functional classification. The literature shows substantial exploratory potential but lacks established evidence of autonomous clinical effectiveness. Most evidence is based on model outputs, simulated cases, expert judgment, or educational outcomes, whereas direct patient-level outcomes and prospective workflow evaluations remain scarce.

The most defensible current role is therefore clinician-supervised assistance: information organization, draft generation, structured feedback, and decision support, with outputs that can be reviewed before they affect care. Greater caution is required for individualized prescription, triage, red-flag interpretation, progression decisions, and direct patient-facing advice. Model and prompt reproducibility, citation and evidence verification, explicit human oversight, and safety monitoring should be treated as core methodological requirements rather than optional reporting details.

Future rehabilitation AI research should prioritize standardized, transparent reporting; prospective multicenter validation; real-world human–AI workflow studies; patient-centered functional outcomes; regulatory evaluation; explainability; and post-deployment safety surveillance. Until such evidence accumulates, generative AI and LLMs should augment—not replace—the contextual clinical judgment of rehabilitation professionals.

## Figures and Tables

**Table 1:** Eligibility criteria defined using the Population–Concept–Context framework.

PCC Element	Inclusion Criteria	Exclusion Criteria
Population	Patients or clients receiving rehabilitation services or undergoing rehabilitation-related assessments; rehabilitation professionals; rehabilitation-related students or trainees; and studies involving real, hypothetical, standardized, virtual, or simulated rehabilitation cases. Rehabilitation-specific datasets, clinical texts, functional data, or exercise/movement data used to evaluate generative AI or LLM applications were also eligible. Relevant fields included physical therapy, occupational therapy, speech-language pathology, physical and rehabilitation medicine/physiatry, rehabilitation counseling, musculoskeletal, neurological, stroke, cardiac, vestibular, sports, and other rehabilitation-related fields.	General patient populations, professionals, students, cases, or datasets unrelated to rehabilitation or functional assessment; studies conducted solely in non-rehabilitation specialties without a rehabilitation-related population, task, case, dataset, or context.
Concept	Studies evaluating generative artificial intelligence, large language models (LLMs), or LLM-based systems applied to rehabilitation-related assessment, interpretation of assessment findings, clinical reasoning, decision support, treatment or rehabilitation planning, goal setting, intervention recommendations, exercise prescription or feedback, functional classification or ICF coding, documentation, professional task support, clinician-directed digital implementation, or rehabilitation education, simulation, and feedback. Prompt-engineering approaches such as zero-shot, few-shot, chain-of-thought, role-play, and retrieval-augmented generation were considered implementation strategies for generative AI and LLMs.	Traditional machine-learning, deep-learning, computer-vision, or rule-based expert-system studies without a generative AI or LLM component; studies using prompting methods without a generative AI/LLM system; generative AI studies unrelated to rehabilitation assessment, functional judgment, clinical reasoning, rehabilitation planning, education, documentation, or decision support.
Context	Rehabilitation-related clinical practice, education, simulation, technical validation, or clinician-directed digital implementation. Eligible contexts included functional and rehabilitation assessment; clinical reasoning; treatment and rehabilitation planning; interpretation of assessment findings; ICF-based classification and documentation; ADL/IADL and functional-status assessment; goal setting; rehabilitation or exercise prescription; exercise-performance feedback; patient-management recommendations; professional task support; virtual or standardized patients; clinical simulations; and rehabilitation-related educational cases.	Administrative, commercial, or general-purpose AI applications unrelated to rehabilitation assessment, functional judgment, rehabilitation planning, education, or decision support; studies limited to general health information, promotional material, or readability of patient-education content without a rehabilitation-specific assessment, reasoning, intervention, educational, or professional-support component.
Types of evidence sources	Peer-reviewed, full-text original empirical studies, including randomized and non-randomized experimental studies, quasi-experimental studies, observational and cross-sectional studies, development and validation studies, performance-evaluation and technical-validation studies, usability or feasibility studies, expert-evaluation studies, case-based studies, simulation studies, and mixed-methods studies.	Systematic reviews, scoping reviews, meta-analyses, umbrella reviews, narrative reviews, protocols, editorials, commentaries, letters, conference abstracts, conference proceedings, preprints, non-peer-reviewed reports, news articles, blogs, commercial promotional materials, duplicate publications, and sources with insufficient information for data extraction.
Language/ date	English-language literature published from 1 January 2015 to 24 July 2026.	Non-English literature and literature published outside the prespecified search period.

**Table 2:** PubMed/MEDLINE search strategy for Generative AI/LLMs and Rehabilitation.

Search Concept	Search Terms
Generative AI/ Large language models	“Large Language Models”[Mesh] OR “large language model”[tiab] OR “large language models”[tiab] OR LLM[tiab] OR LLMs[tiab] OR “generative artificial intelligence”[tiab] OR “generative AI”[tiab] OR GenAI[tiab] OR ChatGPT[tiab] OR “GPT-3.5”[tiab] OR “GPT-4”[tiab] OR “GPT-4o”[tiab] OR “GPT-4.1”[tiab] OR “GPT-5”[tiab] OR “generative pre-trained transformer”[tiab] OR “generative pre-trained transformers”[tiab] OR Gemini[tiab] OR Claude[tiab] OR LLaMA[tiab] OR DeepSeek[tiab] OR Copilot[tiab] OR Perplexity[tiab] OR Mistral[tiab] OR Grok[tiab] OR “foundation model”[tiab] OR “foundation models”[tiab]
Rehabilitation/ Functional and rehabilitation context	“Rehabilitation”[Mesh] OR “Physical Therapy Modalities”[Mesh] OR “Occupational Therapy”[Mesh] OR “Speech-Language Pathology”[Mesh] OR “Physical and Rehabilitation Medicine”[Mesh] OR “Exercise Therapy”[Mesh] OR “Activities of Daily Living”[Mesh] OR “International Classification of Functioning, Disability and Health”[Mesh] OR rehabilitation[tiab] OR rehabilitative[tiab] OR physiotherapy[tiab] OR physiotherapist*[tiab] OR “physical therapy”[tiab] OR “physical therapist*”[tiab] OR “occupational therapy”[tiab] OR “occupational therapist*”[tiab] OR “speech-language pathology”[tiab] OR “speech language pathology”[tiab] OR “speech therapy”[tiab] OR physiatry[tiab] OR “physical and rehabilitation medicine”[tiab] OR “rehabilitation medicine”[tiab] OR neurorehabilitation[tiab] OR “neurological rehabilitation”[tiab] OR “stroke rehabilitation”[tiab] OR “musculoskeletal rehabilitation”[tiab] OR “orthopedic rehabilitation”[tiab] OR “orthopaedic rehabilitation”[tiab] OR “sports rehabilitation”[tiab] OR “cardiac rehabilitation”[tiab] OR “vestibular rehabilitation”[tiab] OR “rehabilitation counseling”[tiab] OR “functional assessment”[tiab] OR “functional outcome*”[tiab] OR “functional status”[tiab] OR ICF[tiab] OR “activities of daily living”[tiab] OR ADL[tiab] OR IADL[tiab] OR “exercise prescription”[tiab] OR “rehabilitation exercise*”[tiab]

* indicates a truncation wildcard used to retrieve different word endings.

**Table 3:** Design-specific methodological appraisal of the studies.

Appraisal Tool	Studies (*n*)	Study Design, Application	Recurrent Strengths	Recurrent Methodological Concerns
MMAT 2018—Quantitative descriptive	31	LLM benchmarking, guideline alignment, expert rating, agreement, and scenario-based quantitative descriptive studies	Structured task definitions, explicit comparison criteria, and quantitative outcome assessment were common.	Case/question sets were often purposive and narrow; some rating instruments were author-developed; conventional nonresponse items fit model-output studies imperfectly.
MMAT 2018—Mixed methods	5	Studies explicitly integrating quantitative and qualitative components	Triangulation of numerical ratings/outcomes with qualitative interpretation.	The integration and handling of discordant qualitative and quantitative findings were not always fully specified, and small sample sizes were common.
JBI Quasi-Experimental Studies (2024)	2	Non-randomized educational intervention or historical/phase comparison	Intervention-comparator structures provided evidence for implementation.	Concurrent controls and repeated measurements were limited, and temporal or cohort confounding remained possible.
JBI Randomized Controlled Trials (2023 revised)	2	Randomized educational/intervention trials	Random allocation and common outcome measurement strengthened causal comparison.	Blinding was infeasible; allocation concealment, baseline balance, attrition, and the analysis approach required caution.
JBI Case Reports	2	Single clinical/test cases	Rich case/task description and transparent clinical context.	Very limited generalizability and no basis for comparative effectiveness.
QUADAS-3 (2026)	1	AI diagnostic/classification validation	Explicit index-test/reference framework and expert annotations.	Curated data and selection/applicability concerns limited transfer to routine clinical populations.

AI, artificial intelligence; JBI, Joanna Briggs Institute; LLM, large language model; MMAT, Mixed Methods Appraisal Tool; QUADAS-3, Quality Assessment of Diagnostic Accuracy Studies-3.

**Table 4:** PCC-based mapping of application domains across the 42 unique studies.

Application Domain	Unique Studies (*n*)	Population	Concept (AI/LLM Application)	Context (Rehabilitation Setting)
Clinical reasoning and decision support	13	Rehabilitation patients or cases, clinicians, and clinical-question datasets	Diagnosis, examination planning, clinical reasoning, triage, treatment decisions, and comparison with clinicians	Musculoskeletal and neurological rehabilitation, sports rehabilitation, occupational therapy, speech-language pathology, and outpatient rehabilitation settings
Rehabilitation education, simulation, and feedback	10	Rehabilitation students, trainees, instructors, and simulated clinical cases	Case simulation, problem-based learning, curriculum support, clinical-interview training, and formative feedback	Physical therapy and occupational therapy education, clinical simulation, fieldwork preparation, and rehabilitation interview training
Rehabilitation planning and prescription	9	Patients, virtual cases, clinical profiles, and expert-generated rehabilitation plans	Generation and evaluation of rehabilitation programs, exercise prescriptions, and individualized treatment plans	Renal, orthopedic, postoperative, cognitive, neurological, and multimorbidity rehabilitation settings
Guideline adherence and clinical question support	6	Guideline-derived questions, clinical scenarios, and rehabilitation professionals	Comparison of AI responses and recommendations with clinical practice guidelines or evidence sources	Musculoskeletal rehabilitation, cardiac rehabilitation, and physical therapy settings involving guideline-based clinical questions
Adaptive feedback and rehabilitation support	2	A patient receiving therapist-mediated intervention and rehabilitation-question datasets	Therapist-facing intervention development and guideline-based agent support	Cognitive rehabilitation and traumatic brain injury rehabilitation using therapist-mediated or guideline-based support
Assessment and functional classification	2	Individuals undergoing postural assessment and curated neuromuscular datasets	Multimodal posture assessment and neuromuscular-state classification using image or waveform inputs	Physiotherapy-based postural assessment and neuromuscular or sports rehabilitation using image and electromyographic data

AI, artificial intelligence; CPG, clinical practice guideline; LLM, large language model; PCC, Population–Concept–Context.

**Table 5:** AI model families evaluated in the 42 unique studies.

AI Model Family	Unique Studies (*n*)	Representative Models	Main Rehabilitation Uses and Prompting/Implementation Strategies
ChatGPT /GPT/ OpenAI GPT family	40	GPT-3.5, GPT-4, GPT-4o, GPT-4.1, GPT-5, OpenAI o3, gpt-oss	Used across most rehabilitation fields for clinical questions, structured case vignettes, diagnostic and treatment reasoning, guideline-based prompts, rehabilitation planning, repeated queries, and limited multimodal or agentic applications.
Gemini family	10	Gemini Advanced, Gemini 2, Gemini 2.5 Flash, Gemini 2.5 Pro	Used for clinical questions, rehabilitation program generation, educational feedback, cross-language prompting, and multimodal comparisons.
DeepSeek family	7	DeepSeek-V2, DeepSeek-V2.5, DeepSeek-V3, DeepSeek-R1	Used mainly in musculoskeletal, postoperative, and rehabilitation-education studies through standardized cases, repeated generations, prompt refinement, and comparative benchmarking.
Claude family	3	Claude 3.7 Sonnet, Claude Opus 4	Used in multispecialty and musculoskeletal rehabilitation for standardized clinical question benchmarking and guideline comparison.
Microsoft Copilot	3	Microsoft Copilot: GPT-4-class implementation reported in one study	Used for rehabilitation program generation, clinical scenarios, and guideline-based question support.
Grok family	3	Grok 3, Grok 4	Used in clinical reasoning and musculoskeletal benchmarking studies with standardized cases and repeated prompting.
Other specialized/regional LLM systems	2	Fine-tuned vision-language systems, Doubao, ERNIE Bot, Qwen, Kimi, Baichuan	Used for multimodal assessment, agentic interpretation, and regional multimodal benchmarking.

AI, artificial intelligence; GPT, generative pre-trained transformer; LLM, large language model.

**Table 6:** Outcome domains and measures across the 42 unique studies.

Outcome Domain	Unique Studies (*n*)	Specific Measures/Constructs	Overall Evidence Pattern
Accuracy, guideline adherence, or agreement with an expert/reference standard	33	Correct-response rate; CPG match/adherence; expert rating; kappa/ICC; consensus/reference agreement.	Performance varied by condition, model, prompt, and reference standard; favorable structured-task performance did not demonstrate broad clinical superiority.
Clinical reasoning, decision quality, treatment planning, or prescription quality	25	Case understanding; examination planning; reasoning; treatment-plan accuracy/completeness/relevance/applicability.	LLMs often produced plausible structure but retained limitations in complex reasoning, contextual integration, specificity, and individualized judgment.
Consistency, repeatability, or reliability	11	Repeated-query stability; response consistency; kappa/ICC; comparable reliability metrics.	Only a subset directly tested output stability; consistency varied across models, prompts, tasks, and rater frameworks.
Safety, clinical appropriateness, applicability, or risk-sensitive evaluation	11	Safety ratings; red flags; contraindications; applicability; inappropriate content; treatment-plan safety.	Safety was not uniformly assessed; clinically significant outputs still required professional review.
Educational outcomes, feedback perception, or learner experience	10	Knowledge/proficiency; Mini-CEX; self-efficacy; engagement; learner perceptions; feedback preference/quality.	Some educational effects were favorable, but the transfer to independent clinical competence and patient outcomes remains uncertain.
Personalization, specificity, progression, or real-world applicability of rehabilitation plans	8	Individualization; dosage/frequency; progression; feasibility; goal orientation; need for expert correction.	Generated plans frequently required revisions to dosage, progression, specificity, feasibility, or integration with the patient’s context.
Feasibility, usability, implementation, or acceptability	8	Use/uptake; satisfaction; acceptability; implementation burden/cost; educational/clinical fit.	Feasibility was generally promising, but actual uptake, resource assumptions, and the implementation context varied.
Technical classification, automation, multimodal assessment, or agent performance	3	Classification/consensus performance; multimodal assessment; agent responsiveness/explainability.	Technical feasibility was demonstrated, but prospective external clinical validation remained limited.
Reference/evidence reliability, hallucination, fabrication, or falsification	2	Reference existence/appropriateness; unsupported/fabricated content; evidentiary reliability.	Citation/evidence reliability remained a safety concern but was directly assessed in only a few studies.
Actual patient-level clinical/functional outcome after an LLM-assisted intervention	1	Patient-level functional and clinical outcomes after a therapist-mediated LLM-assisted intervention.	Only one case report directly assessed a patient-level outcome; therefore, clinical effectiveness remains unestablished.

CPG, clinical practice guideline; ICC, intraclass correlation coefficient; LLM, large language model; Mini-CEX, Mini-Clinical Evaluation Exercise. Outcome-domain counts are not mutually exclusive and may therefore exceed 42 when summed. Companion publications derived from the same underlying cohort were consolidated, and each unique study was counted once within each outcome domain.

## Data Availability

Study-level extraction, methodological appraisal, model/prompt/reproducibility characteristics, outcomes, limitations, and funding data are presented in the supplementary tables accompanying this manuscript.

## Supplementary Materials

The following supporting information can be downloaded at: https://www.mdpi.com/article/10.3390/life16081310/s1, Table S1. Complete search strategies for all databases; Table S2. Study-level methodological appraisal and source-based explanations for non-favorable items; Table S3. General characteristics of studies; Table S4. Study-level model, version, prompting, access, and reproducibility characteristics; Table S5. Study-level tasks, outcomes, main findings, safety/clinical limitations, and funding/financial support; Table S6. Operational coding framework and ambiguity-resolution rules for the six application domains; Table S7. Preferred Reporting Items for Systematic reviews and Meta-Analyses extension for Scoping Reviews (PRISMA-ScR) Checklist.
