# Supplementary material for: Generative AI and Large Language Models in Rehabilitation: A Scoping Review
Source: Life (Basel). 2026 Aug 10;16(8):1310. doi: 10.3390/life16081310 (PMC13514507; doi:10.3390/life16081310)
Supplement: Supplementary file 1 [file life-16-01310-s001.zip › life-4458277-supplementary.pdf]

Supplementary Table S1. Complete search strategies for all databases.

Databases	Search Strategies
PubMed/ MEDLINE	("Large Language Models"[Mesh] OR "large language model"[tiab] OR "large language models"[tiab] OR LLM[tiab] OR LLMs[tiab] OR "generative artificial intelligence"[tiab] OR "generative AI"[tiab] OR GenAI[tiab] OR ChatGPT[tiab] OR "GPT-3.5"[tiab] OR "GPT-4"[tiab] OR "GPT-4o"[tiab] OR "GPT-4.1"[tiab] OR "GPT-5"[tiab] OR "generative pre-trained transformer"[tiab] OR "generative pre-trained transformers"[tiab] OR Gemini[tiab] OR Claude[tiab] OR LLaMA[tiab] OR DeepSeek[tiab] OR Copilot[tiab] OR Perplexity[tiab] OR Mistral[tiab] OR Grok[tiab] OR "foundation model"[tiab] OR "foundation models"[tiab]) AND ("Rehabilitation"[Mesh] OR "Physical Therapy Modalities"[Mesh] OR "Occupational Therapy"[Mesh] OR "Speech-Language Pathology"[Mesh] OR "Physical and Rehabilitation Medicine"[Mesh] OR "Exercise Therapy"[Mesh] OR "Activities of Daily Living"[Mesh] OR "International Classification of Functioning, Disability and Health"[Mesh] OR rehabilitation[tiab] OR rehabilitative[tiab] OR physiotherapy[tiab] OR physiotherapist[tiab] OR physiotherapists[tiab] OR "physical therapy"[tiab] OR "physical therapist"[tiab] OR "physical therapists"[tiab] OR "occupational therapy"[tiab] OR "occupational therapist"[tiab] OR "occupational therapists"[tiab] OR "speech-language pathology"[tiab] OR "speech language pathology"[tiab] OR "speech therapy"[tiab] OR physiatry[tiab] OR "physical and rehabilitation medicine"[tiab] OR "rehabilitation medicine"[tiab] OR neurorehabilitation[tiab] OR "neurological rehabilitation"[tiab] OR "stroke rehabilitation"[tiab] OR "musculoskeletal rehabilitation"[tiab] OR "orthopedic rehabilitation"[tiab] OR "orthopaedic rehabilitation"[tiab] OR "sports rehabilitation"[tiab] OR "cardiac rehabilitation"[tiab] OR "vestibular rehabilitation"[tiab] OR "rehabilitation counseling"[tiab] OR "functional assessment"[tiab] OR "functional outcome"[tiab] OR "functional outcomes"[tiab] OR "functional status"[tiab] OR ICF[tiab] OR "activities of daily living"[tiab] OR ADL[tiab] OR IADL[tiab] OR "exercise prescription"[tiab] OR "rehabilitation exercise"[tiab] OR "rehabilitation exercises"[tiab])
CINAHL	XB ("large language model" OR "large language models" OR LLM OR LLMs OR "generative artificial intelligence" OR "generative AI" OR GenAI OR ChatGPT OR "GPT-3.5" OR "GPT-4" OR "GPT-4o" OR "GPT-4.1" OR "GPT-5" OR "generative pre-trained transformer" OR "generative pre-trained transformers" OR Gemini OR Claude OR LLaMA OR DeepSeek OR Copilot OR Perplexity OR Mistral OR Grok OR "foundation model" OR "foundation models") AND XB (rehabilitat* OR physiotherap* OR "physical therapy" OR "physical therapist" OR "physical therapists" OR "occupational therapy" OR "occupational therapist" OR "occupational therapists" OR "speech-language pathology" OR "speech language pathology" OR "speech therapy" OR physiatry OR "physical and rehabilitation medicine" OR "rehabilitation medicine" OR neurorehabilitation OR "neurological rehabilitation" OR "stroke rehabilitation" OR "musculoskeletal rehabilitation" OR "orthopedic rehabilitation" OR "orthopaedic rehabilitation" OR "sports rehabilitation" OR "cardiac rehabilitation" OR "vestibular rehabilitation" OR "rehabilitation counseling" OR "functional assessment" OR "functional outcome" OR "functional outcomes" OR "functional status" OR ICF OR "International Classification of Functioning, Disability and Health" OR "activities of daily living" OR ADL OR IADL OR "exercise prescription" OR "rehabilitation exercise" OR "rehabilitation exercises")
Web of Science Core Collection	("large language model*" OR LLM* OR "generative artificial intelligence" OR "generative AI" OR GenAI OR ChatGPT OR "GPT-3.5" OR "GPT-4*" OR "GPT-5" OR "generative pre-trained transformer*" OR Gemini OR Claude OR LLaMA OR DeepSeek OR Copilot OR Perplexity OR Mistral OR Grok OR "foundation model*") AND (rehabilitat* OR physiotherap* OR "physical therapy" OR "physical therapist*" OR "occupational therap*" OR "speech-language patholog*" OR "speech language patholog*" OR "speech therap*" OR physiatry OR "physical and rehabilitation medicine" OR "rehabilitation medicine" OR neurorehabilitation OR "neurological rehabilitation" OR "stroke rehabilitation" OR "musculoskeletal rehabilitation" OR "orthopedic rehabilitation" OR "orthopaedic rehabilitation" OR "sports rehabilitation" OR "cardiac rehabilitation" OR "vestibular rehabilitation" OR "rehabilitation counseling" OR "functional assessment" OR "functional outcome*" OR "functional status" OR ICF OR "International Classification of Functioning, Disability and Health" OR "activities of daily living" OR ADL OR IADL OR "exercise prescription" OR "rehabilitation exercise*")
Scopus	TITLE-ABS-KEY("large language model" OR "large language models" OR LLM OR LLMs OR "generative artificial intelligence" OR "generative AI" OR GenAI OR ChatGPT OR "GPT-3.5" OR "GPT-4" OR "GPT-4o" OR "GPT-4.1" OR "GPT-5" OR "generative pre-trained transformer" OR "generative pre-trained transformers" OR Gemini OR Claude OR LLaMA OR DeepSeek OR Copilot OR Perplexity OR Mistral OR Grok OR "foundation model" OR "foundation models") AND TITLE-ABS-KEY(rehabilitat* OR physiotherap* OR "physical therapy" OR "physical therapist" OR "physical therapists" OR "occupational therapy" OR "occupational therapist" OR "occupational therapists" OR "speech-language pathology" OR "speech language pathology" OR "speech therapy" OR physiatry OR "physical and rehabilitation medicine" OR "rehabilitation medicine" OR neurorehabilitation OR "neurological rehabilitation" OR "stroke rehabilitation" OR "musculoskeletal rehabilitation" OR "orthopedic rehabilitation" OR "orthopaedic rehabilitation" OR "sports rehabilitation" OR "cardiac rehabilitation" OR "vestibular rehabilitation" OR "rehabilitation counseling" OR "functional assessment" OR "functional outcome" OR "functional outcomes" OR "functional status" OR ICF OR "International Classification of Functioning, Disability and Health" OR "activities of daily living" OR ADL OR IADL OR "exercise prescription" OR "rehabilitation exercise" OR "rehabilitation exercises") AND PUBYEAR > 2014 AND PUBYEAR < 2027 AND LANGUAGE(english)
IEEE Xplore	((Search_All:"large language model" OR "All Metadata":"large language models" OR "All Metadata":LLM OR "All Metadata":LLMs OR "All Metadata":"generative artificial intelligence" OR "All Metadata":"generative AI" OR "All Metadata":GenAI OR "All Metadata":ChatGPT OR "All Metadata":"GPT-3.5" OR "All Metadata":"GPT-4" OR "All Metadata":"GPT-4o" OR "All Metadata":"GPT-4.1" OR "All Metadata":"GPT-5" OR "All Metadata":"generative pre-trained transformer" OR "All Metadata":"generative pre-trained transformers" OR "All Metadata":Gemini OR "All Metadata":Claude OR "All Metadata":LLaMA OR "All Metadata":DeepSeek OR "All Metadata":Copilot OR "All Metadata":Perplexity OR "All Metadata":Mistral OR "All Metadata":Grok OR "All Metadata":"foundation model" OR "All Metadata":"foundation models") AND ((Search_All:rehabilitat* OR "All Metadata":physiotherap* OR "All Metadata":"physical therapy" OR "All Metadata":"physical therapist" OR

Databases	Search Strategies
	"All Metadata":"physical therapists" OR "All Metadata":"occupational therapy" OR "All Metadata":"occupational therapist" OR "All Metadata":"occupational therapists" OR "All Metadata":"speech-language pathology" OR "All Metadata":"speech language pathology" OR "All Metadata":"speech therapy" OR "All Metadata":physiatry OR "All Metadata":"physical and rehabilitation medicine" OR "All Metadata":"rehabilitation medicine" OR "All Metadata":neurorehabilitation OR "All Metadata":"neurological rehabilitation" OR "All Metadata":"stroke rehabilitation" OR "All Metadata":"musculoskeletal rehabilitation") OR (Search_All:"orthopedic rehabilitation" OR "All Metadata":"orthopaedic rehabilitation" OR "All Metadata":"sports rehabilitation" OR "All Metadata":"cardiac rehabilitation" OR "All Metadata":"vestibular rehabilitation" OR "All Metadata":"rehabilitation counseling" OR "All Metadata":"functional assessment" OR "All Metadata":"functional outcome" OR "All Metadata":"functional outcomes" OR "All Metadata":"functional status" OR "All Metadata":ICF OR "All Metadata":"International Classification of Functioning, Disability and Health" OR "All Metadata":"activities of daily living" OR "All Metadata":ADL OR "All Metadata":IADL OR "All Metadata":"exercise prescription" OR "All Metadata":"rehabilitation exercise" OR "All Metadata":"rehabilitation exercises")))

Database-specific syntax, indexing terms, search fields, truncation symbols, and filters were adapted to the requirements of each database. The search covered English-language literature published from 1 January 2015 to 24 July 2026.

Supplementary Table S2. Study-level methodological appraisal and source-based explanations for non-favorable items.

Study	Verified design	Appraisal tool	Item/domain result	Non-favorable / non-Y item(s)	Source-based explanation
Bandara et al. (2026) [19]	AI diagnostic/classification validation study	QUADAS-3 (2026) – with applicability caution	P=High; I=Unclear; T=Low; A=Unclear; App(P/I/T)=High concern/Unclear concern/Low concern	QUADAS-3 Participants=High; Index test=Unclear; Analysis=Unclear; Participants applicability=High concern; Index-test applicability=Unclear concern	The index test used a curated H-reflex EMG image dataset with expert annotations rather than a consecutive or random clinical sample. This raises concerns about participant selection and applicability; some index-test/analysis details were insufficient to definitively judge low risk.
Bezruk et al. (2026) [20]	Cross-language comparative experimental model-output study	MMAT 2018 – Quantitative descriptive	Y, N, CT, CT, Y	MMAT-QD Q2=N, Q3=CT, Q4=CT	The case/question/evaluator set was narrow or purposively selected, limiting the representativeness of the target rehabilitation domain. At least part of the evaluation rubric/measurement framework was study-specific or lacked clear external validation. The conventional nonresponse item had limited direct fit to model-output benchmarking and was therefore coded conservatively.
Cao et al. (2026) [21]	Convergent mixed-methods benchmarking study	MMAT 2018 – Mixed methods	Y, Y, Y, CT, Y	MMAT-MM Q4=CT	Both quantitative and qualitative components were relevant, but integration procedures and explicit handling of discordant findings were not always fully reported; therefore, judgments regarding mixed-methods integration and component quality were conservative.
Cheng et al. (2026) [22]	Comparative expert-rating quantitative study	MMAT 2018 – Quantitative descriptive	Y, N, CT, CT, Y	MMAT-QD Q2=N, Q3=CT, Q4=CT	The case/question/evaluator set was narrow or purposively selected, limiting the representativeness of the target rehabilitation domain. At least part of the evaluation rubric/measurement framework was study-specific or lacked clear external validation. The conventional nonresponse item had limited direct fit to model-output benchmarking and was therefore coded conservatively.
Dahò & Caci (2026) [23]	Mixed-methods evaluation	MMAT 2018 – Mixed methods	Y, Y, Y, CT, CT	MMAT-MM Q4=CT, Q5=CT	Both quantitative and qualitative components were relevant, but integration procedures and

Study	Verified design	Appraisal tool	Item/domain result	Non-favorable / non-Y item(s)	Source-based explanation
					explicit handling of discordant findings were not always fully reported; therefore, judgments regarding mixed-methods integration and component quality were conservative.
Değerli et al. (2026) [24]	Comparative quantitative descriptive study	MMAT 2018 – Quantitative descriptive	Y, N, CT, CT, Y	MMAT-QD Q2=N, Q3=CT, Q4=CT	The case/question/evaluator set was narrow or purposively selected, limiting the representativeness of the target rehabilitation domain. At least part of the evaluation rubric/measurement framework was study-specific or lacked clear external validation. The conventional nonresponse item had limited direct fit to model-output benchmarking and was therefore coded conservatively.
Garcia et al. (2026) [25]	Cross-sectional guideline-alignment study	MMAT 2018 – Quantitative descriptive	Y, CT, Y, CT, Y	MMAT-QD Q2=CT, Q4=CT	The representativeness of the selected cases, questions, and participants for the wider target domain was not fully established. The conventional nonresponse item has limited direct fit to model-output benchmarking and was therefore coded conservatively.
Hamrin Senorski et al. (2026) [26]	Comparative benchmarkin g / quantitative descriptive	MMAT 2018 – Quantitative descriptive	Y, CT, Y, CT, Y	MMAT-QD Q2=CT, Q4=CT	The representativeness of the selected cases, questions, and participants for the wider target domain was not fully established. The conventional nonresponse item has limited direct fit to model-output benchmarking and was therefore coded conservatively.
Kaya et al. (2026) [27]	Comparative expert-rating quantitative study	MMAT 2018 – Quantitative descriptive	Y, CT, Y, CT, Y	MMAT-QD Q2=CT, Q4=CT	The representativeness of the selected cases, questions, and participants for the wider target domain was not fully established. The conventional nonresponse item has limited direct fit to model-output benchmarking and was therefore coded conservatively.
Knauer et al. (2026) [28]	Convergent mixed-methods pilot study	MMAT 2018 – Mixed methods	Y, Y, Y, CT, Y	MMAT-MM Q4=CT	Both quantitative and qualitative components were relevant, but integration procedures and explicit handling of discordant findings were not always fully reported; therefore, judgments regarding mixed-methods integration and component quality were conservative.
Liu et al. (2026) [29]	LLM performance benchmarkin g / quantitative descriptive	MMAT 2018 – Quantitative descriptive	Y, CT, Y, CT, Y	MMAT-QD Q2=CT, Q4=CT	The representativeness of the selected cases, questions, and participants for the wider target domain was not fully established. The conventional nonresponse item has limited direct fit to model-output benchmarking and was therefore coded conservatively.
Luo et al. (2026) [30]	Prompt-engineering comparative performance study	MMAT 2018 – Quantitative descriptive	Y, CT, Y, CT, Y	MMAT-QD Q2=CT, Q4=CT	The representativeness of the selected cases, questions, and participants for the wider target domain was not fully established. The conventional nonresponse item has limited direct fit to model-output

Study	Verified design	Appraisal tool	Item/domain result	Non-favorable / non-Y item(s)	Source-based explanation
					benchmarking and was therefore coded conservatively.
Michou et al. (2026) [31]	Cross-sectional comparative educational performance study	MMAT 2018 – Quantitative descriptive	Y, CT, Y, Y, Y	MMAT-QD Q2=CT	The representativeness of the selected cases, questions, and participants for the wider target domain was not fully established.
Sortino et al. (2026) [32]	Observational cross-sectional comparative study	MMAT 2018 – Quantitative descriptive	Y, CT, CT, CT, Y	MMAT-QD Q2=CT, Q3=CT, Q4=CT	The representativeness of the selected cases, questions, and participants for the wider target domain was not fully established. At least part of the evaluation rubric or measurement framework was study-specific or lacked clear external validation. The conventional nonresponse item had limited direct fit to model-output benchmarking and was therefore coded conservatively.
Sozlu et al. (2026) [33]	Cross-sectional agreement study	MMAT 2018 – Quantitative descriptive	Y, CT, Y, Y, Y	MMAT-QD Q2=CT	The representativeness of the selected cases, questions, and participants for the wider target domain was not fully established.
Tuncer et al. (2026) [34]	Comparative quantitative performance study	MMAT 2018 – Quantitative descriptive	Y, N, CT, CT, Y	MMAT-QD Q2=N, Q3=CT, Q4=CT	The case/question/evaluator set was narrow or purposively selected, limiting the representativeness of the target rehabilitation domain. At least part of the evaluation rubric/measurement framework was study-specific or lacked clear external validation. The conventional nonresponse item had limited direct fit to model-output benchmarking and was therefore coded conservatively.
Villagrán et al. (2026) [35]	Within-cohort two-phase quasi-experimental educational study	JBI Quasi-Experimental 2024 revised	Y, N, Y, U, N, Y, Y, Y, Y	JBI-QE Q2=N, Q4=U, Q5=N	Two phases were compared without a concurrent control. Time/phase effects could not be fully separated from the AI-feedback intervention, and repeated pre- and post-measurements within each phase were limited.
Arbel et al. (2025) [36]	Comparative performance study / quantitative descriptive	MMAT 2018 – Quantitative descriptive	Y, CT, Y, Y, Y	MMAT-QD Q2=CT	The representativeness of the selected cases, questions, and participants for the wider target domain was not fully established.
Bezruk et al. (2025) [37]	Descriptive AI-modeling / literature-comparison study	MMAT 2018 – Quantitative descriptive	Y, N, CT, CT, Y	MMAT-QD Q2=N, Q3=CT, Q4=CT	The case/question/evaluator set was narrow or purposively selected, limiting the representativeness of the target rehabilitation domain. At least part of the evaluation rubric/measurement framework was study-specific or lacked clear external validation. The conventional nonresponse item had limited direct fit to model-output benchmarking and was therefore coded conservatively.
Birol et al. (2025) [38]	Cross-sectional descriptive expert-rating study	MMAT 2018 – Quantitative descriptive	Y, N, CT, Y, Y	MMAT-QD Q2=N	The case/question/evaluator set was narrow or purposively selected, limiting the representativeness of the target rehabilitation domain.
Değerli YI & Özata	Case report	JBI Case Reports	Y, Y, Y, Y, Y, Y, N, Y	JBI-CR Q7=N	Single MCI case with a detailed assessment/intervention course.

Study	Verified design	Appraisal tool	Item/domain result	Non-favorable / non-Y item(s)	Source-based explanation
Değerli (2025) [39]					Adverse or unexpected events were not systematically reported, limiting the corresponding JBI case-report item.
Ergezen Sahin et al. (2025) [40]	Randomized controlled trial	JBI RCT 2023 revised	Y, U, N, N, N, Y, U, Y, Y, Y, N, Y, Y	JBI-RCT Q2=U, Q3=N, Q4=N, Q5=N, Q7=U, Q11=N	Randomization was reported, but allocation concealment and assessor blinding were not fully described; participant/intervention-provider blinding was infeasible in an educational trial. Baseline/analysis issues were conservatively judged where reporting was incomplete.
Ferrer-Peña et al. (2025) [41]	Pilot randomized parallel-group study	JBI RCT 2023 revised	Y, Y, N, N, N, Y, Y, Y, N, Y, N, Y, Y	JBI-RCT Q3=N, Q4=N, Q5=N, Q9=N, Q11=N	The pilot used a randomized design with external randomization, but educational blinding was infeasible. Baseline balance, low actual LLM uptake, and analysis by randomized assignment required caution.
Geneş et al. (2025) [42]	Cross-sectional descriptive performance study	MMAT 2018 – Quantitative descriptive	Y, CT, Y, CT, Y	MMAT-QD Q2=CT, Q4=CT	The representativeness of the selected cases, questions, and participants for the wider target domain was not fully established. The conventional nonresponse item has limited direct fit to model-output benchmarking and was therefore coded conservatively.
Gürses et al. (2025a) [43]	Observational comparative quantitative study	MMAT 2018 – Quantitative descriptive	Y, CT, CT, CT, Y	MMAT-QD Q2=CT, Q3=CT, Q4=CT	The representativeness of the selected cases, questions, and participants for the wider target domain was not fully established. At least part of the evaluation rubric or measurement framework was study-specific or lacked clear external validation. The conventional nonresponse item had limited direct fit to model-output benchmarking and was therefore coded conservatively.
Gürses et al. (2025b) [44]	Observational comparative quantitative study	MMAT 2018 – Quantitative descriptive	Y, CT, Y, CT, Y	MMAT-QD Q2=CT, Q4=CT	The representativeness of the selected cases, questions, and participants for the wider target domain was not fully established. The conventional nonresponse item has limited direct fit to model-output benchmarking and was therefore coded conservatively.
Hao et al. (2025) [45]	Simulation/case-based quantitative performance evaluation	MMAT 2018 – Quantitative descriptive	Y, CT, Y, CT, Y	MMAT-QD Q2=CT, Q4=CT	The representativeness of the selected cases, questions, and participants for the wider target domain was not fully established. The conventional nonresponse item has limited direct fit to model-output benchmarking and was therefore coded conservatively.
Inoue et al. (2025) [46]	Expert-rating quantitative descriptive study	MMAT 2018 – Quantitative descriptive	Y, N, CT, Y, Y	MMAT-QD Q2=N, Q3=CT	The case/question/evaluator set was narrow or purposively selected, limiting the representativeness of the target rehabilitation domain. At least part of the evaluation rubric or measurement framework was study-specific or lacked clear external validation.

Study	Verified design	Appraisal tool	Item/domain result	Non-favorable / non-Y item(s)	Source-based explanation
Lee & Park (2025) [47]	Expert-rating quantitative descriptive study	MMAT 2018 – Quantitative descriptive	Y, N, CT, Y, Y	MMAT-QD Q2=N, Q3=CT	The case/question/evaluator set was narrow or purposively selected, limiting the representativeness of the target rehabilitation domain. At least part of the evaluation rubric or measurement framework was study-specific or lacked clear external validation.
Marcaccini et al. (2025) [48]	Experimental scenario-based quantitative performance study	MMAT 2018 – Quantitative descriptive	Y, N, Y, Y, Y	MMAT-QD Q2=N	The case/question/evaluator set was narrow or purposively selected, limiting the representativeness of the target rehabilitation domain.
Mykhalko et al. (2025) [49]	Expert-evaluated quantitative descriptive study	MMAT 2018 – Quantitative descriptive	Y, N, CT, Y, Y	MMAT-QD Q2=N, Q3=CT	The case/question/evaluator set was narrow or purposively selected, limiting the representativeness of the target rehabilitation domain. At least part of the evaluation rubric or measurement framework was study-specific or lacked clear external validation.
Okuno et al. (2025) [50]	Non-randomized historical-control quasi-experimental study	JBI Quasi-Experimental 2024 revised	Y, Y, Y, U, N, Y, Y, Y, Y	JBI-QE Q4=U, Q5=N	The historical-control design provided a comparator but remained vulnerable to cohort and time effects; there were no repeated outcome measurements around the intervention.
Rossettini et al. (2025) [51]	Guideline-adherence quantitative descriptive study	MMAT 2018 – Quantitative descriptive	Y, CT, Y, CT, Y	MMAT-QD Q2=CT, Q4=CT	The representativeness of the selected cases, questions, and participants for the broader target domain was not fully established. The conventional nonresponse item has limited direct alignment with model-output benchmarking and was therefore coded conservatively.
Safran & Yildirim (2025) [52]	Guideline-alignment quantitative descriptive study	MMAT 2018 – Quantitative descriptive	Y, CT, Y, CT, Y	MMAT-QD Q2=CT, Q4=CT	The representativeness of the selected cases, questions, and participants for the broader target domain was not fully established. The conventional nonresponse item has limited direct alignment with model-output benchmarking and was therefore coded conservatively.
Saglam et al. (2025) [53]	Cross-sectional comparative performance study	MMAT 2018 – Quantitative descriptive	Y, CT, Y, CT, Y	MMAT-QD Q2=CT, Q4=CT	The representativeness of the selected cases, questions, and participants for the wider target domain was not fully established. The conventional nonresponse item has limited direct fit to model-output benchmarking and was therefore coded conservatively.
Sudo et al. (2025) [54]	Mixed-methods study	MMAT 2018 – Mixed methods	Y, Y, Y, CT, CT	MMAT-MM Q4=CT, Q5=CT	Both quantitative and qualitative components were relevant, but integration procedures and explicit handling of discordant findings were not always fully reported; therefore, judgments about mixed-methods integration and component quality were conservative.
Zhang et al. (2025) [55]	Guideline-adherence quantitative descriptive study	MMAT 2018 – Quantitative descriptive	Y, CT, Y, CT, Y	MMAT-QD Q2=CT, Q4=CT	The representativeness of the selected cases, questions, and participants for the wider target domain was not fully established. The conventional

Study	Verified design	Appraisal tool	Item/domain result	Non-favorable / non-Y item(s)	Source-based explanation
					nonresponse item has limited direct fit to model-output benchmarking and was therefore coded conservatively.
Zou et al. (2025) [56]	Multicenter cross-sectional agreement study	MMAT 2018 – Quantitative descriptive	Y, Y, Y, Y, Y	No non-favorable items; all MMAT-QD items = Y	The study met the main design-specific criteria, with remaining limitations captured in the item-level judgments shown.
Bilika et al. (2024) [57]	Case-vignette performance evaluation / quantitative descriptive	MMAT 2018 – Quantitative descriptive	Y, N, CT, CT, Y	MMAT-QD Q2=N, Q3=CT, Q4=CT	The case/question/evaluator set was narrow or purposively selected, limiting the representativeness of the target rehabilitation domain. At least part of the evaluation rubric/measurement framework was study-specific or lacked clear external validation. The conventional nonresponse item had limited direct fit to model-output benchmarking and was therefore coded conservatively.
Mansour & Wong (2024) [58]	Mixed-methods sequential/convergent educational study	MMAT 2018 – Mixed methods	Y, Y, CT, CT, CT	MMAT-MM Q3=CT, Q4=CT, Q5=CT	The study combined pre- and post-quantitative survey data with qualitative/open-ended responses. The integration and handling of divergent findings were not fully explicit, and the quantitative component had attrition (34 pre, 27 post); therefore, the mixed-methods integration and component-quality items were conservatively judged as CT.
Sawamura et al. (2024) [59]	Quantitative descriptive accuracy study	MMAT 2018 – Quantitative descriptive	Y, CT, Y, CT, Y	MMAT-QD Q2=CT, Q4=CT	The representativeness of the selected cases, questions, and participants for the wider target domain was not fully established. The conventional nonresponse item has limited direct fit to model-output benchmarking and was therefore coded conservatively.
Zhenzhu et al. (2024) [60]	Non-human comparative quantitative performance study	MMAT 2018 – Quantitative descriptive	Y, N, Y, CT, Y	MMAT-QD Q2=N, Q4=CT	The case/question/evaluator set was narrow or purposively selected, limiting the representativeness of the target rehabilitation domain. The conventional nonresponse item had limited direct fit to model-output benchmarking and was therefore coded conservatively.
Zhang et al. (2023) [61]	Comparative single case report	JBI Case Reports	Y, Y, Y, Y, Y, N/A, N/A, Y	JBI-CR Q6=N/A, Q7=N/A	This was a comparative textbook test case rather than a real treated patient; therefore, post-intervention clinical status and adverse-event items were not applicable.

CT, cannot tell; JBI, Joanna Briggs Institute; MMAT, Mixed Methods Appraisal Tool; N, no; NA, not applicable; QUADAS-3, Quality Assessment of Diagnostic Accuracy Studies-3; Y, yes. Item-level appraisal is presented without a summed quality score or exclusion threshold.

Supplementary Table S3. General characteristics of studies.

Study	Country	Study design	Rehabilitation field /context	Application domain	AI model(s)
Bandara et al. (2026) [19]	USA-led / international	AI diagnostic, classification validation study	Neuromuscular / sports rehabilitation	Assessment / functional classification	OpenAI GPT-OSS reasoning LLM + fine-tuned VLM consortium (exact GPT-

Study	Country	Study design	Rehabilitation field /context	Application domain	AI model(s)
					OSS variant NR)
Bezruk et al. (2026) [20]	Ukraine	Cross-language comparative model-output study	Renal rehabilitation / CKD	Rehabilitation planning / prescription	Gemini 2.5 Flash; GPT-4.1; Microsoft Copilot (GPT-4-class, authors' label)
Cao et al. (2026) [21]	China	Convergent mixed-methods benchmarking study	Rehabilitation medicine (multispecialty)	Clinical reasoning / decision support	Grok-4; Gemini-2.5-Pro; ChatGPT-5-2025-08-07; DeepSeek-R1-0528; Claude-Opus-4-20250514
Cheng et al. (2026) [22]	China	Comparative expert rating study	Postoperative / breast cancer rehabilitation	Rehabilitation planning / prescription	ChatGPT (version NR); DeepSeek (version NR)
Dahò & Caci (2026) [23]	Italy	Mixed-methods evaluation	Cognitive / executive-function rehabilitation	Rehabilitation planning/prescription	ChatGPT-4o
Değerli et al. (2026) [24]	Türkiye	Comparative study	Occupational therapy	Clinical reasoning / decision support	ChatGPT-3.5; free public interface
Garcia et al. (2026) [25]	USA	Cross-sectional guideline-alignment study	Musculoskeletal / knee osteoarthritis	Guideline-concordant clinical support	GPT-3.5; GPT-4o
Hamrin Senorski et al. (2026) [26]	Sweden-led international	Comparative benchmarking study	Sports rehabilitation	Clinical reasoning / decision support	GPT-4 via ChatGPT
Kaya et al. (2026) [27]	Türkiye	Comparative expert-evaluation study	Sports injury rehabilitation	Clinical reasoning / decision support	GPT-4o; GPT-5
Knauer et al. (2026) [28]	Germany / Austria	Convergent mixed-methods pilot study	Musculoskeletal / low-back pain physiotherapy	Clinical reasoning / decision support	Gemini 2.5 Pro; OpenAI o3; DeepSeek-R1-0528
Liu et al. (2026) [29]	China	LLM benchmarking / clinical decision-support evaluation	Musculoskeletal rehabilitation	Clinical reasoning / decision support	ChatGPT-4.1; Grok 3; Claude 3.7 Sonnet; Gemini 2.5 Flash Thinking; DeepSeek-R1; Doubao 1.5 Pro; ERNIE Bot X1 Turbo; Qwen 3.0; Kimi 1.5; Baichuan 4 Turbo
Luo et al. (2026) [30]	China	Prompt-engineering comparative performance study	Musculoskeletal / low-back pain	Clinical reasoning / decision support	DeepSeek-V2.5; GPT-5; GPT-4o; OpenAI o3; Grok-4
Michou et al. (2026) [31]	Greece	Cross-sectional comparative educational performance study	Physiotherapy education / clinical rehabilitation	Education / simulation / feedback	GPT-4.1; DeepSeek-V2
Sortino et al. (2026) [32]	Italy	Observational cross-sectional comparative study	Orthopedic rehabilitation / adapted physical activity	Rehabilitation planning / prescription	Generative AI model / version NR in verified source
Sozlu et al. (2026) [33]	Türkiye	Cross-sectional agreement study	Postural assessment / physiotherapy	Assessment / functional classification	ChatGPT-5.1 multimodal
Tuncer et al. (2026) [34]	Türkiye	Prospective observational comparative study	Spinal neurosurgery / physical therapy	Clinical reasoning / decision support	GPT-4; GPT-3.5
Villagrán et al. (2026) [35]	Chile	Within-cohort two-phase quasi-experimental educational study	Physiotherapy education / clinical interview training	Education / simulation / feedback	GPT-4 embedded in a local AI-supported feedback platform
Arbel et al. (2025) [36]	Israel	Comparative cross-sectional educational performance study	Vestibular rehabilitation education	Education/simulation/feedback	ChatGPT-3.5 (2023 version); Google Gemini (2024 version); free interfaces
Bezruk et al. (2025)	Ukraine	Descriptive AI-modeling / literature-	Renal rehabilitation /	Rehabilitation planning/prescription	Gemini; ChatGPT (exact versions NR)

Study	Country	Study design	Rehabilitation field /context	Application domain	AI model(s)
[37]		comparison study	nephrology		
Birol et al. (2025) [38]	Türkiye	Cross-sectional descriptive / expert-evaluation study	Speech-language pathology	Clinical reasoning / decision support	ChatGPT-4.0
Değerli YI & Özata Değerli (2025) [39]	Türkiye	Case report	Occupational therapy / cognitive rehabilitation	Adaptive feedback / rehabilitation support	ChatGPT (exact version NR)
Ergezen Sahin et al. (2025) [40]	Türkiye / Spain	Randomized controlled trial	Physiotherapy education	Education / simulation / feedback	ChatGPT-4.0
Ferrer-Peña et al. (2025) [41]	Spain	Pilot randomized parallel-group feasibility study	Physiotherapy education	Education / simulation / feedback	Article states ChatGPT 3.5 participant accounts; cost section references ChatGPT Team Plan v4.0—runtime model not fully unambiguous
Geneş et al. (2025) [42]	Türkiye	Cross-sectional guideline / clinical-scenario evaluation	Cardiac rehabilitation	Guideline-concordant clinical support	ChatGPT-4o (methods/body; abstract uses "ChatGPT-4")
Gürses et al. (2025a) [43]	Türkiye	Observational comparative study	Musculoskeletal / knee osteoarthritis	Rehabilitation planning / prescription	ChatGPT-4o; Gemini Advanced
Gürses et al. (2025b) [44]	Türkiye	Observational comparative study	Musculoskeletal / knee osteoarthritis	Rehabilitation planning / prescription	ChatGPT-4o; Gemini Advanced
Hao et al. (2025) [45]	USA / China	Simulation-based performance evaluation	Musculoskeletal physiotherapy education	Education / simulation / feedback	ChatGPT-4
Inoue et al. (2025) [46]	Japan	Expert-rating feasibility study	Occupational therapy / stroke rehabilitation	Rehabilitation planning / prescription	ChatGPT/GPT (exact version NR in verified source)
Lee & Park (2025) [47]	Republic of Korea	Expert-evaluation educational study	Neurological occupational therapy education	Education / simulation / feedback	ChatGPT (exact version NR)
Marcaccini et al. (2025) [48]	Australia / Italy-led	Scenario-based multi-model expert evaluation	Head and neck postoperative rehabilitation	Rehabilitation planning / prescription	ChatGPT-4o; DeepSeek V3; Gemini 2; Microsoft Copilot
Mykhalko et al. (2025) [49]	Ukraine	Cross-sectional expert evaluation	Physical rehabilitation / multimorbidity	Rehabilitation planning / prescription	ChatGPT-4o
Okuno et al. (2025) [50]	Japan	Non-randomized historical-control quasi-experimental study	Physiotherapy education / internal disorders	Education / simulation / feedback	ChatGPT (GPT-4)
Rossettini et al. (2025) [51]	Italy-led international	Observational cross-sectional guideline-adherence study	Musculoskeletal / lumbosacral radicular pain	Guideline-concordant clinical support	ChatGPT-3.5; ChatGPT-4o; Microsoft Copilot; Google Gemini; Claude; Perplexity
Safran & Yildirim (2025) [52]	Türkiye	Cross-sectional observational guideline-alignment study	Musculoskeletal rehabilitation	Guideline-concordant clinical support	ChatGPT (GPT-4 model)
Saglam et al. (2025) [53]	Türkiye	Cross-sectional comparative study	Sports surgery / physiotherapy	Clinical reasoning / decision support	GPT-4; GPT-3.5
Sudo et al. (2025) [54]	Japan	Mixed-methods comparative educational study	Physiotherapy education	Education / simulation / feedback	Gemini 2.5 Pro
Zhang et al. (2025) [55]	China	Cross-sectional guideline-adherence study	Musculoskeletal / plantar fasciitis	Guideline-concordant clinical support	ChatGPT-4o; ChatGPT-4 Turbo
Zou et al. (2025) [56]	China	Multicenter cross-sectional agreement study	Outpatient rehabilitation triage	Clinical reasoning / decision support	ChatGPT-4 via a Chinese network portal
Bilika et	Greece	Case-based clinical	Physiotherapy /	Clinical reasoning /	ChatGPT-3.5; free

Study	Country	Study design	Rehabilitation field /context	Application domain	AI model(s)
al. (2024) [57]		reasoning performance study	musculoskeletal clinical reasoning	decision support	interface
Mansour & Wong (2024) [58]	USA	One-group pre-post educational study	Occupational-therapy education / fieldwork readiness	Education / simulation / feedback	ChatGPT v3.5
Sawamura et al. (2024) [59]	Japan	Cross-sectional response/ reference accuracy study	Physical therapy / multi-condition	Guideline-concordant clinical support	ChatGPT free version (exact backend version NR)
Zhenzhu et al. (2024) [60]	China	Non-human comparative technical/performance study	Traumatic brain injury rehabilitation	Adaptive feedback / rehabilitation support	GPT-4; guideline-based GPT agents
Zhang et al. (2023) [61]	Japan	Comparative single-case test	Physical medicine and rehabilitation / stroke	Clinical reasoning / decision support	ChatGPT-4

AI, artificial intelligence; AWS, axillary web syndrome; CKD, chronic kidney disease; LLM, large language model; NR, not reported; OT, occupational therapy; PT, physical therapy. Model names are reproduced or standardized only to the extent explicitly supported by each source.

Supplementary Table S4. Study-level model, version, prompting, access, and reproducibility characteristics.

Study	Provider/ model(s)/ exact version	Interface/ API/ deployment	Access date	Prompting/ implementation strategy	Full prompt/ template available	Repeated runs	Generation settings	Retrieval /AG / external tool use	Output variability/ consistency handling	Comparator/ reference standard/ evaluators
Bandara et al. (2026) [19]	OpenAI GPT-OSS reasoning LLM + fine-tuned VLM consortium (exact GPT-OSS variant NR)	Model pipeline; exact deployment interface NR	NR	Prompt-engineered multimodal/agentic reasoning pipeline using H-reflex EMG images	NR	NR	NR	External tool/agent pipeline reported	NR	Expert clinical annotations and recovery/athlete metadata
Bezruk et al. (2026) [20]	Gemini 2.5 Flash; GPT-4.1; Microsoft Copilot (GPT-4-class, authors' label)	Separate model sessions; same device/browser/network; chat history cleared	Date/time documented in article Supplementary Table S1 (exact timestamp not reproduced in the main text)	One standardized prompt in equivalent Ukrainian and English versions; no iterative refinement	Full prompts provided in Supplementary File S1	Single attempt per condition/language/model	Default configurations; no temperature/randomness adjustment available beyond platform defaults	NR	Single-attempt design; language effects compared across models	Clinical guideline alignment and structured content comparison
Cao et al. (2026) [21]	Grok-4; Gemini-2.5-Pro; ChatGPT-5-2025-08-07; DeepSeek-R1-0528; Claude-Opus-4-20250514	Official model APIs/web interfaces	10 August 2025	Standardized clinical-case/role prompt across 48 cases	Full English prompt reported in the appendix	NR	Temperature = 0.7; no task-specific fine-tuning	NR	NR	Expert-authored plans; six senior physiatrists blinded to source

Study	Provider/ model(s)/ exact version	Interface/ API/ deployment	Access date	Prompting/ implementation strategy	Full prompt/ template available	Repeated runs	Generation settings	Retrieval /AG / external tool use	Output variability/ consistency handling	Comparator/ reference standard/ evaluators
Cheng et al. (2026) [22]	ChatGPT (version NR); DeepSeek (version NR)	NR	NR	Standardized case-based prompts across five virtual postoperative AWS cases	Full wording NR	NR	NR	NR	NR	Three rehabilitation specialists; rubric covering diagnosis, comprehensiveness, individualization, safety, evidence, clarity
Dahò & Caci (2026) [23]	ChatGPT-4o	Browser-based ChatGPT-4o; no logged-in account; two independent devices/IPs/ browsers	23 June 2025 and 7 July 2025	Expert-generated and refined prompts for three ADHD profiles; outputs evaluated using the ISAAC framework	Prompt framework/materials reported	Each profile prompt entered twice (6 plans generated total)	NR	NR	Independent duplicate generation plus 27 external experts	17 prompt-development experts and 27 external experts
Değerli et al. (2026) [24]	ChatGPT-3.5; free public interface	Free public ChatGPT interface	NR	Role-based OT instruction plus nine structured questions across three clinical scenarios; new chats used	Prompt/task structure reported	Multiple interactions/new sessions used; exact repeated-run count NR	NR	NR	111 occupational therapists as comparison group	111 occupational therapists
Garcia et al. (2026) [25]	GPT-3.5; GPT-4o	NR	NR	Common knee-OA treatment questions benchmarked against four CPGs	Prompt/question set reported in article/materials	NR	NR	NR	Two independent reviewers plus adjudication for guideline matching	Four CPGs selected using AGREE II; two independent reviewers and adjudication
Hamrin Senorski et al. (2026) [26]	GPT-4 via ChatGPT	Online ChatGPT	NR	Structured sports rehabilitation prompts tailored to patient, PT, and expert-PT audiences	NR	NR	NR	NR	Three senior raters blinded to response source	Three senior physiotherapist/researcher panel, blinded to response source
Kaya et al. (2026) [27]	GPT-4o; GPT-5	ChatGPT web interface; separate sessions	NR	Standardized zero-shot prompt; identical scenario input for GPT-4o and GPT-5	NR	Three runs per scenario/ model	Default system-controlled settings; no fine-tuning reported	No RAG used/reported	Repeated outputs were used to assess consistency and reliability	Two blinded consistency raters; five orthopedic specialists scoring clinical adequacy
Knauer et al.	Gemini 2.5 Pro;	Model-specific	OpenAI o3: 2	Standardized low-	NR	Five identical	Gemini: temperatu	NR	Test-retest semantic	Expert rating

Study	Provider/ model(s)/ exact version	Interface/ API/ deployment	Access date	Prompting/ implementation strategy	Full prompt/ template available	Repeated runs	Generation settings	Retrieval /AG / external tool use	Output variability/ consistency handling	Comparator/ reference standard/ evaluators
(2026) [28]	OpenAI o3; DeepSeek-R1-0528	online/service interfaces	Jul 2025; DeepSeek-R1-0528: 3 Jul 2025; Gemini 2.5 Pro: 4 Jul 2025	back-pain case-vignette prompts		inputs per task/case for test-retest analysis	re 0; OpenAI o3: default 1; DeepSeek-R1-0528: default/temperature ignored in inference		similarity explicitly assessed	framework plus qualitative analysis
Liu et al. (2026) [29]	ChatGPT-4.1; Grok 3; Claude 3.7 Sonnet; Gemini 2.5 Flash Thinking; DeepSeek-R1; Doubao 1.5 Pro; ERNIE Bot X1 Turbo; Qwen 3.0; Kimi 1.5; Baichuan 4 Turbo	Online model interfaces/services reported	Exact model names/versions reported; query date NR in accessible text	Structured musculoskeletal-rehabilitation examination/case prompts	NR	NR	NR	NR	Comparative model benchmarking	Five senior doctors/therapists as assessors
Luo et al. (2026) [30]	DeepSeek-V2.5; GPT-5; GPT-4o; OpenAI o3; Grok-4	Model interfaces/services NR	NR	Baseline clinical prompts followed by structured prompt-engineering remediation emphasizing safety, evidence, uncertainty, and actionability	Prompt-remediation framework reported	Clinical questions/scenarios were generated three times per model in the reported evaluation	NR	NR	Repeated generation used for stability and performance analyses	Guideline-informed and expert-reviewed evaluation framework
Michou et al. (2026) [31]	GPT-4.1; DeepSeek-V2	NR	NR	Sixty standardized clinical questions across four rehabilitation topics	NR	NR	NR	NR	NR	Study scoring framework for the 60 questions
Sortino et al. (2026) [32]	Generative AI model/version NR in verified source	NR	NR	Identical standardized prompt used for AI-assisted and AI-alone prescription conditions	NR	NR	NR	NR	NR	Multidisciplinary panel (n=135)
Sozlu et al.	ChatGPT-5.1	ChatGPT web interface	15-16 Novem	Standardized multimod	NR	NR	NR	NR	Regional agreement with two	Two physiotherapists using a

Study	Provider/ model(s)/ exact version	Interface/ API/ deployment	Access date	Prompting/ implementation strategy	Full prompt/ template available	Repeated runs	Generation settings	Retrieval /AG / external tool use	Output variability/ consistency handling	Comparator/ reference standard/ evaluators
(2026) [33]	multimodal		ber 2025	al posture-evaluation prompt with photographs					physiotherapists	seven-region posture form
Tuncer et al. (2026) [34]	GPT-4; GPT-3.5	NR	NR	Ten standardized detailed spinal clinical scenarios	NR	NR	NR	NR	64 clinician/academic evaluators	64 clinician/academic evaluators
Villagrán et al. (2026) [35]	GPT-4 embedded in a local AI-supported feedback platform	Local AI-supported feedback platform	NR	GPT-4 used on the platform to score, rationalize, and summarize instructor feedback; system prompt NR	NR	NR	NR	NR	Phase comparison of instructor feedback quality	Instructor feedback quality criteria and phase comparison
Arbel et al. (2025) [36]	ChatGPT-3.5 (2023 version); Google Gemini (2024 version); free interfaces	Free ChatGPT and Google Gemini interfaces	NR	Same 20-item Vestibular Knowledge Test submitted to both models; no additional training or prompt refinement reported	Question set reported	One response per question /model reported	NR	NR	Expert accuracy ratings from three board-certified otoneurologists	Professionals/students plus three board-certified otoneurologists
Bezruk et al. (2025) [37]	Gemini; ChatGPT (exact versions NR)	NR	NR	Rehabilitation program generation prompts for nephrology patients; compared with published exercise complexes	Exact wording NR	NR	NR	NR	Literature-based content comparison	Published rehabilitation literature/systematic evidence
Birol et al. (2025) [38]	ChatGPT-4.0	ChatGPT interface; exact deployment NR	10-11 July 2023	Structured SLP task prompts across six core tasks and seven disorder areas	Task prompts reported/ described	One recorded response per prompt reported	NR	NR	15 practicing SLPs rated accuracy, relevance, and comprehensiveness	15 practicing speech-language pathologists
Değerli .YI & Özata Değerli (2025) [39]	ChatGPT (exact version NR)	Therapist-facing ChatGPT use; exact interface NR	NR	Therapist-directed development of two simple computerized	NR	NR	NR	NR	Clinical pre/post case assessments	Clinical assessments and pre/post case outcome

Study	Provider/ model(s)/ exact version	Interface/ API/ deployment	Access date	Prompting/ implementation strategy	Full prompt/ template available	Repeated runs	Generation settings	Retrieval /AG / external tool use	Output variability/ consistency handling	Comparator/ reference standard/ evaluators
				assistive intervention programs; patient did not directly interact with ChatGPT						
Ergezen Sahin et al. (2025) [40]	ChatGPT-4.0	NR	June 2025 intervention period; exact model query date NR	Supervised ChatGPT-4.0-supported PBL case/problem interaction	Educational procedure reported; complete model prompt set NR	Not a repeated model benchmark	NR	NR	Traditional PBL randomized control group	Traditional PBL control group
Ferrer-Peña et al. (2025) [41]	Article states ChatGPT 3.5 participant accounts; cost section references ChatGPT Team Plan v4.0—runtime model not fully unambiguous	Participant ChatGPT accounts; article reports v3.5 accounts, while cost section references Team Plan v4.0	Study Sep 2023–Jan 2024; exact query dates NR	Four weekly clinical cases using predefined prompts	Predefined prompts shown/reported in article	Students could query cases repeatedly; exact per-case run counts were not reported	NR	NR	Randomized parallel-group feasibility comparison	Randomized parallel-group comparison
Geneş et al. (2025) [42]	ChatGPT-4o (methods/ body; abstract uses "ChatGPT-4")	Publicly available ChatGPT interface; exact deployment NR	NR	40 independent prompts (20 general open-ended + 20 clinical scenarios); ChatGPT restarted between questions	Questions provided in appendices	One recorded response per independent prompt	NR	NR	Two expert evaluators; guideline-based categorization	Cardiac rehabilitation guidelines/expert evaluation
Gürses et al. (2025a) [43]	ChatGPT-4o; Gemini Advanced	ChatGPT and Gemini interfaces; exact deployment NR	NR	Patient assessment data submitted in Turkish; models asked to create individualized three-phase exercise programs	Prompt structure reported	One program per patient/ model reported	NR	NR	Association of 12 assessment parameters with recommendations	Patient assessment data and statistical associations of 12 parameters with model recommendations
Gürses et al. (2025b) [44]	ChatGPT-4o; Gemini Advanced	ChatGPT and Gemini interfaces; new chat sessions per patient	Aug–Oct 2024 study period; exact	Structured Turkish patient-specific prompts; English	Prompt available (article/supplement)	One individualized program per	NR	NR	Consensus of three physiotherapists	Consensus of three physiotherapists

Study	Provider/ model(s)/ exact version	Interface/ API/ deployment	Access date	Prompting/ implementation strategy	Full prompt/ template available	Repeated runs	Generation settings	Retrieval /AG / external tool use	Output variability/ consistency handling	Comparator/ reference standard/ evaluators
			query dates NR	translation in the article; original Turkish prompt in the supplement		patient/ model				
Hao et al. (2025) [45]	ChatGPT-4	NR	NR	Sequential SOAP-based staged prompting; clinical data revealed incrementally	Full instructional prompt and S/O/A/P questions printed in the article	One staged response sequence per case	NR	NR	Two DPT raters; consensus scores	Study rubric/reference answers for 10 cases
Inoue et al. (2025) [46]	ChatGPT/ GPT (exact version NR in verified source)	NR	NR	Case-based OT rehabilitation program generation for five stroke cases with psychological symptoms	NR	NR	NR	NR	Five occupational therapists	Five occupational therapists
Lee & Park (2025) [47]	ChatGPT (exact version NR)	NR	NR	Neurological OT case-generation prompts	NR	NR	NR	NR	Ten expert raters	Ten expert raters
Marcaccini et al. (2025) [48]	ChatGPT-4o; DeepSeek V3; Gemini 2; Microsoft Copilot	Online model interfaces; exact deployment NR	NR	Identical standardized scenario prompt across 10 head-and-neck postoperative cases	Verbatim core prompt reported in article	One response per scenario/ model reported	NR	NR	Clinician expert ratings	Clinician expert ratings
Mykhalko et al. (2025) [49]	ChatGPT-4o	ChatGPT interface; exact deployment NR	NR	Prompted to create a one-month individualized physical rehabilitation plan for each of 50 comorbid cases	Prompt requirement described; full template NR	One plan per case reported	NR	NR	Two rehabilitation experts; six-criterion rubric and inter-rater reliability	Two calibrated rehabilitation experts; six-criterion rubric
Okuno et al. (2025) [50]	ChatGPT (GPT-4)	ChatGPT/GPT-4; exact interface NR	NR	LLM-assisted development of longitudinal, respiratory, story-centered curriculum cases	Example prompts reported in article	Curriculum development; repeated generation count NR	NR	NR	Historical-control cohort	Historical control cohort

Study	Provider/ model(s)/ exact version	Interface/ API/ deployment	Access date	Prompting/ implementation strategy	Full prompt/ template available	Repeated runs	Generation settings	Retrieval /AG / external tool use	Output variability/ consistency handling	Comparator/ reference standard/ evaluators
Rossettini et al. (2025) [51]	ChatGPT-3.5; ChatGPT-4o; Microsoft Copilot; Google Gemini; Claude; Perplexity	Public chatbot interfaces (latest 2024 versions)	NR	Nine CPG-derived clinical questions submitted to six chatbots	Question set reported	Repeated queries were performed for text-consistency assessment; exact run count should be taken from the source	NR	NR	Text consistency plus intra/inter-rater reliability and CPG match	Clinical practice guideline recommendations; inter-observer classification
Safran & Yildirim (2025) [52]	ChatGPT (GPT-4 model)	ChatGPT interface; each question in a new session	NR	Twenty CPG-based musculoskeletal questions; model instructed to answer from a physiotherapist's perspective	Questions reported	One session per question; separate-session design	NR	NR	Two raters; five quality dimensions and weighted kappa	Two experienced MSK physiotherapists and CPGs
Saglam et al. (2025) [53]	GPT-4; GPT-3.5	NR	NR	Ten standardized sports-surgery/physiotherapy scenarios for GPT-4 and GPT-3.5	Scenario framework reported	NR	NR	NR	56 professionals rated four clinical domains	Expert/structured evaluation criteria
Sudo et al. (2025) [54]	Gemini 2.5 Pro	Web-based Gemini 2.5 Pro interface	July 2025	Zero-shot versus few-shot prompting; the few-shot condition included 9 teacher-created examples	User prompts and example prior feedback available in article(s)/supplement	No repeated benchmark procedure reported	Platform default settings, including default temperature; no manual parameter adjustments	NR	35 students; FPQ, preference, and focus-group evaluation	Student feedback-quality/perception measures and qualitative data
Zhang et al. (2025) [55]	ChatGPT-4o; ChatGPT-4 Turbo	NR	NR	Guideline-based plantar fasciitis management questions	Question set reported	NR	NR	NR	Accuracy, consistency, self-awareness, and fabrication/falsification ratings	Evidence-based clinical practice guidelines
Zou et al. (2025) [56]	ChatGPT-4 via a Chinese network portal	ChatGPT-4 connected via a Chinese network portal	NR	Sequential patient triage information supplied with the model configured as an experienc	NR	One classification per patient reported	NR	NR	300 patients; DRP tool and rehabilitation doctors	Outpatient rehabilitation doctors and DRP tool comparisons

Study	Provider/ model(s)/ exact version	Interface/ API/ deployment	Access date	Prompting/ implementation strategy	Full prompt/ template available	Repeated runs	Generation settings	Retrieval /AG / external tool use	Output variability/ consistency handling	Comparator/ reference standard/ evaluators
				ed rehabilitation doctor in China						
Bilika et al. (2024) [57]	ChatGPT-3.5; free interface	Free ChatGPT interface	NR	Repeated identical PT assessment question plus published simple/complex case scenarios	Question/case prompts reported	Study 1: 10 independent submissions/users; Study 2: two independent users	NR	NR	Consistency, omissions, and reasoning compared across users/cases	Expected physiotherapy assessment content and published case information
Mansour & Wong (2024) [58]	ChatGPT v3.5	ChatGPT v3.5 interface; exact deployment NR	NR	Assignment-based generation of OT intervention ideas within a two-week educational activity	Assignment procedure reported	Not a repeated model benchmark	NR	NR	Pre/post student survey plus qualitative responses	Pre/post student survey
Sawamura et al. (2024) [59]	ChatGPT free version (exact backend version NR)	Free ChatGPT interface	3 April 2023	15 guideline-derived Japanese PT clinical questions (5 each: stroke, musculoskeletal, internal disorders) with requests for PMID/DOI references	Exact standardized prompt printed in article	One response per question reported	NR	PubMed/DOI verification used for references	Six raters (two per section); weighted kappa	Japanese PT guidelines, PubMed/DOI verification, and two expert assessors
Zhenzhu et al. (2024) [60]	GPT-4; guideline-based GPT agents	GPT-4 plus guideline-based multi-agent system	NR	Direct GPT-4 compared with guideline-enhanced agent prompts for 30 TBI rehabilitation questions	Agent framework/prompting described	NR	NR	Retrieval/guideline classification, question retrieval, matching, QA, evaluation, and source-citation agents	Responsiveness, explainability, and answer-quality comparison	Medical rehabilitation guidelines / study evaluation framework
Zhang et al. (2023) [61]	ChatGPT-4	ChatGPT-4 interface; exact deployment NR	NR	Case-based queries for medical/rehabilitation problems, management, goals, PT/OT/SLT, assistive devices,	Prompt details provided in Appendix S1	Single textbook test case	NR	NR	Textbook standard answer plus two PM&R clinicians	Textbook case solution and two PM&R clinicians

Study	Provider/ model(s)/ exact version	Interface/ API/ deployment	Access date	Prompting/ implementation strategy	Full prompt/ template available	Repeated runs	Generation settings	Retrieval /AG / external tool use	Output variability/ consistency handling	Comparator/ reference standard/ evaluators
				and ICF codes						

API, application programming interface; CPG, clinical practice guideline; DPT, Doctor of Physical Therapy; LLM, large language model; NR, not reported; OT, occupational therapy; PBL, problem-based learning; RAG, retrieval-augmented generation; SLP, speech-language pathologist; SOAP, Subjective–Objective–Assessment–Plan. “NR” indicates that the item was not explicitly reported in the verified source; no value was inferred.

Supplementary Table S5. Study-level tasks, outcomes, main findings, safety/clinical limitations, and funding/financial support.

Study	Primary application	Task evaluated	Main outcome / measure	Main finding	Safety / clinical limitation	Funding/financial support
Bandara et al. (2026) [19]	Assessment / functional classification	Classify neuromuscular states (fatigue, injury, recovery) from H-reflex EMG waveform images and refine interpretation with a reasoning LLM.	Classification/consensus performance and interpretability	The multimodal consortium-plus-reasoning LLM produced highly accurate, consistent, and interpretable classifications on the curated dataset.	Curated, annotated data rather than a prospective, representative clinical cohort; external clinical validity and generalizability remain uncertain.	None
Bezruk et al. (2026) [20]	Rehabilitation planning / prescription	Generate rehabilitation programs for CKD/hemodialysis and compare the output structure, clinical detail, and guideline alignment across query languages.	Language-related differences in structure, clinical specificity, and guideline alignment	English prompts consistently produced more detailed, clinically structured, and guideline-aligned programs; the query language influenced the output more than the model did. Copilot was the most clinically precise among the evaluated systems.	No prospective patient-level validation; output quality was language-sensitive.	None
Cao et al. (2026) [21]	Clinical reasoning / decision support	Generate rehabilitation management plans and compare them with expert-authored plans through multidimensional blinded evaluation.	Clinical applicability/safety, scientific rigor, individualization, clarity; thematic differences	Grok-4 and Gemini-2.5-Pro received the highest overall expert ratings, while experts maintained an advantage in strategic pathway design and humanistic care. Top LLMs were useful for preliminary regimen generation rather than for replacing specialists.	Safety/applicability was explicitly scored, but evaluation remained case-based rather than prospective workflow or patient-outcome validation.	Medical–Engineering Interdisciplinary Research Program of the First Affiliated Hospital of the University of South China (IRP-M&E-2025-09); Clinical Research 4310 Program of the First Affiliated Hospital of the University of South China (20214310NHYCG07)
Cheng et al. (2026) [22]	Rehabilitation planning / prescription	Generate assessment and rehabilitation recommendations across cases of varying complexity.	Expert-rated diagnostic and rehabilitation-plan quality	DeepSeek scored significantly higher overall, including diagnostic accuracy (p=.006), comprehensiveness (p=.034), and individualization (p=.005). ChatGPT	Only five virtual cases; exploratory design with no real-world patient validation.	None

Study	Primary application	Task evaluated	Main outcome / measure	Main finding	Safety / clinical limitation	Funding/financial support
				responses were more generalized and sometimes omitted differential diagnostic considerations.		
Dahò & Caci (2026) [23]	Rehabilitation planning / prescription	Develop expert-informed prompts, generate ChatGPT-based executive-function rehabilitation plans, and evaluate their validity, relevance, and feasibility.	Theoretical validity, clinical relevance, feasibility, and qualitative plan characteristics	Plans were viewed as conceptually and theoretically promising, especially for adolescent and adult profiles, but feasibility concerns included insufficient personalization, unrealistic resource assumptions, and some unvalidated techniques.	Hypothetical profiles and no patient trials; feasibility and personalization limitations require expert adaptation.	None
Değerli et al. (2026) [24]	Clinical reasoning / decision support	Compare ChatGPT and OT approaches to models/frames of reference, assessment, and intervention across case scenarios.	Agreement/differences in clinical decision-making domains	ChatGPT and therapists made similar decisions in one of three cases within each major domain but differed in the others, highlighting limitations in individualized and context-sensitive professional reasoning.	Case-based comparison; contextual and individualized reasoning remained dependent on occupational therapists.	None
Garcia et al. (2026) [25]	Guideline-concordant clinical support	Compare ChatGPT's treatment information with recommendations from high-quality clinical practice guidelines for knee OA.	Thematic agreement with guideline recommendations	Across 10 themes and 33 subthemes, 6.06% showed high agreement, 18.18% moderate agreement, and 75.75% low agreement with the selected CPGs.	Low guideline agreement was predominant; clinicians should critically verify AI-generated treatment information.	NR
Hamrin Senorski et al. (2026) [26]	Clinical reasoning / decision support	Answer sports-rehabilitation questions at the patient, physical therapist, and expert physical therapist levels.	Response quality and adaptiveness	GPT-4 significantly outperformed junior expert physical therapists overall ($p<.001$) and was rated best on 55% of patient-targeted and 64% of physiotherapist-targeted questions.	Controlled question-and-answer setting; no evidence that superiority in expert ratings translates into real-world patient outcomes.	None
Kaya et al. (2026) [27]	Clinical reasoning / decision support	Compare clinical responses from GPT-4o and GPT-5 across standardized sports-injury scenarios.	Consistency, clinical adequacy and inter-rater reliability	GPT-4o showed 93.3% consistency (κ = .82). GPT-5 received a higher median clinical adequacy score than GPT-4o (4.60 vs. 4.00; p = .007); inter-rater ICCs were .888 and .895.	Scenario-based expert evaluation does not establish the safety of autonomous diagnosis or treatment.	None
Knauer et al. (2026) [28]	Clinical reasoning / decision support	Evaluate reasoning-model responses to physiotherapy case vignettes	Reasoning scores, inter-rater reliability, test-retest semantic similarity and qualitative themes	Models were generally rated as competent or proficient in conceptual reasoning, with no	Red-flag prioritization, empathy, patient-centeredness, and clinical	Physio Deutschland; German Federal Ministry of Education and Research (BMBF;

Study	Primary application	Task evaluated	Main outcome / measure	Main finding	Safety / clinical limitation	Funding/financial support
		across conceptual, therapeutic, and human-centered dimensions.		clear differences between models. Test-retest semantic similarity was high (.90-.94), but inter-rater reliability was poor overall (ICC = .12). Qualitative analysis highlighted rigid, checklist-like reasoning and weak intuition, empathy, and shared decision-making.	intuition remained weak; the pilot sample limits generalizability.	16DHBKI071); German Research Foundation (DFG; 528483508); OA publication support from St. Pölten University of Applied Sciences
Liu et al. (2026) [29]	Clinical reasoning / decision support	Assess medical competence and clinical decision support for rehabilitation across six dimensions.	Case understanding, reasoning, diagnosis/differential diagnosis, treatment-plan accuracy/safety, guideline-consensus use	In the first test, ERNIE Bot X1 Turbo and Doubao 1.5 Pro were the only models exceeding 90% accuracy; Chinese LLMs made fewer incorrect responses than the evaluated English-language LLMs. The study also examined the use of LLM assistance in therapist examination preparation.	Performance varied substantially across models and test dimensions; benchmark competence does not establish safe independent clinical use.	Department of Science and Technology of Hubei Province (2024BCB007); National Key Research and Development Program of China (2022YFB4700200); Natural Science Foundation of Hubei Province (2024AFB958)
Luo et al. (2026) [30]	Clinical reasoning / decision support	Diagnose LLM clinical-reasoning errors and test whether structured prompt engineering improves performance.	Five-dimensional reasoning performance and pre/post prompt-remediation change	The study identified systematic reasoning deficits in low-back-pain management and showed that structured prompt engineering could improve selected performance dimensions, although clinically relevant reasoning errors persisted.	Prompt improvement did not eliminate errors; red-flag prioritization and reliable transfer to clinical care remain concerns.	Investigator-Initiated Clinical Research Program of the Pudong New Area Health Commission (2025-PWYC-11)
Michou et al. (2026) [31]	Education / simulation / feedback	Compare student and LLM responses to questions about rehabilitation knowledge, diagnosis, alternatives, and rehabilitation.	Written-response quality across four clinical topics and question types	The evaluated LLMs generally produced higher-quality written responses than final-year students on the standardized questions, but the study did not establish equivalence to qualified clinicians or real-world competence.	Educational benchmark only; student comparisons should not be interpreted as evidence for autonomous patient management.	None
Sortino et al. (2026) [32]	Rehabilitation planning / prescription	Compare adapted physical-activity prescriptions from expert-professional, novice-plus-AI, and AI-alone.	Effectiveness, safety, appropriateness, clarity and progression	Expert-professional prescriptions ranked highest, novice-plus-AI intermediate, and AI-alone lowest. Experts	AI-alone prescriptions were weakest in safety, tailoring, and progression; expert oversight significantly	None

Study	Primary application	Task evaluated	Main outcome / measure	Main finding	Safety / clinical limitation	Funding/financial support
				outperformed AI across all conditions (p<.01), with the largest gaps in safety, tailoring, and progression.	improved quality.	
Sozlu et al. (2026) [33]	Assessment / functional classification	Evaluate standardized posture photographs using a multimodal ChatGPT model and compare regional classifications with those of physiotherapists.	Regional agreement/kappa with expert assessment	Agreement was highest for the trunk (κ = .85) and the knee (κ = .83), moderate to substantial for the head-neck (κ = .67), and lower for the scapular, shoulder, pelvic, and foot regions (κ = .43–.51); overall agreement ranged from 64.10% to 92.31%.	More reliable for gross deviations than for fine angular discrimination; photographic AI assessment should supplement rather than replace physical examination.	None
Tuncer et al. (2026) [34]	Clinical reasoning / decision support	Compare GPT-4 and GPT-3.5 for clinical decision-making across diagnosis, treatment, surgical details, and rehabilitation planning.	Multi-criteria expert scores	GPT-4 significantly outperformed GPT-3.5 overall (t(63) = 18.00, p < .001); treatment suitability and rehabilitation plan evaluations also favored GPT-4 (F = 60.53 and F = 57.55, respectively).	Scenario-based ratings rather than patient outcomes; superior model performance does not establish autonomous safety.	OA publication support from the Scientific and Technological Research Council of Türkiye (TÜBİTAK)
Villagrán et al. (2026) [35]	Education / simulation / feedback	Compare instructor feedback before and after the introduction of an AI-supported feedback platform.	Direct observation, specificity, positive reinforcement, action planning, and suggestions for improvement	The AI-supported phase improved direct observation, specific information, positive reinforcement, and action planning (all p<.01). The AI augmented instructor feedback rather than replacing instructors.	Educational implementation only; effectiveness depends on instructor mediation and context.	Chilean National Agency for Research and Development (ANID; Doctorado Becas Chile/2020-21202032); National Center for Artificial Intelligence (CENIA; FB210017 BASAL); Pontificia Universidad Católica de Chile (FONDEDOC-2022)
Arbel et al. (2025) [36]	Education / simulation / feedback	Compare professionals, students, and two LLMs on vestibular knowledge, basic practice, and clinical reasoning, and independently rate LLM answer accuracy.	VKT score and expert-rated answer accuracy	ChatGPT scored 70%, and Gemini scored 60%. Both scored 100% on Clinical Knowledge but only 50% and 25%, respectively, on Clinical Reasoning. ChatGPT responses were deemed comprehensive for 45% of questions and completely incorrect for 25%.	Clinical reasoning was substantially weaker than factual knowledge; the source advises cautious integration with clinical expertise and guidelines.	None
Bezruk et al. (2025) [37]	Rehabilitation planning / prescription	Develop physical exercise programs for nephrology patients and	Content and clinical relevance of AI-generated exercise programs	AI-generated programs covered respiratory, aerobic, strength, stretching, and	Descriptive modeling without prospective patient	NR

Study	Primary application	Task evaluated	Main outcome / measure	Main finding	Safety / clinical limitation	Funding/financial support
		compare their content with published rehabilitation evidence.		relaxation components and were considered potentially useful for routine support, but their quality depended on clinician expertise and evidence checking.	validation; generated content requires expert review.	
Birol et al. (2025) [38]	Clinical reasoning / decision support	Evaluate ChatGPT responses across common SLP professional tasks and disorders.	Accuracy, comprehensiveness, relevance, and professional usefulness	Responses were predominantly rated highly for accuracy, comprehensiveness, and relevance, whereas therapy stimuli and detailed therapy planning showed greater variability.	Professional task support may be useful, but treatment planning still requires expert judgment specific to the domain.	OA publication support from the Scientific and Technological Research Council of Türkiye (TÜBİTAK)
Değerli .Yl & Özata Değerli (2025) [39]	Adaptive feedback / rehabilitation support	Use ChatGPT as a therapist-facing development resource for two simple computer-based assistive-intervention programs.	ADL performance/independence and case feasibility	The therapist used ChatGPT to support the development of intervention materials; the case reported improved ADL performance and independence after the 8-week intervention.	Single case with no control; ChatGPT served as a therapist support tool, not an autonomous patient-facing intervention.	None
Ergezen Sahin et al. (2025) [40]	Education / simulation / feedback	Compare AI-supported problem-based learning with traditional PBL for developing clinical reasoning.	Theoretical knowledge, Mini-CEX, AI self-efficacy, internet addiction, reading motivation; pre/post and follow-up	The randomized trial reported educational benefits of the AI-supported approach for selected learning and clinical-reasoning outcomes, but interpretation is tempered by the small sample size and the unavoidable lack of blinding in the educational intervention.	Small educational RCT; no patient outcomes.	None
Ferrer-Peña et al. (2025) [41]	Education / simulation / feedback	Test the feasibility of using LLM-based clinical cases for training in clinical reasoning.	Feasibility, engagement, satisfaction, and implementation cost	Recruitment and participation were high, but only 5 of 23 participants assigned to the LLM group actively used the tool as intended. Overall satisfaction did not differ significantly; the reported initial implementation cost was US$1,738. The article contains an internally inconsistent percentage next to the 5/23 fraction, so the fraction is reported here.	Pilot feasibility study; low uptake limits conclusions about educational effectiveness.	Publication support from La Salle Center for Higher University Studies (LCHUS)

Study	Primary application	Task evaluated	Main outcome / measure	Main finding	Safety / clinical limitation	Funding/financial support
Geneş et al. (2025) [42]	Guideline-concordant clinical support	Assess ChatGPT-4o responses to 40 guideline-informed cardiac rehabilitation questions (20 general open-ended and 20 clinical scenarios).	Guideline compliance and adequacy	Among general questions, 14/20 were fully compliant, and six were compliant but insufficient; among scenarios, 16/20 were fully compliant, and four were compliant but insufficient. Strengths included risk stratification and safety, with less detail for older adults and HIIT.	Incomplete details in some clinically important contexts; responses require expert verification.	None
Gürses et al. (2025a) [43]	Rehabilitation planning / prescription	Generate three-phase exercise programs from patient assessment data and test whether recommendations varied by clinical parameters.	Parameter-sensitive exercise recommendations across three phases	ChatGPT-4o showed significant differences in recommendations across seven assessment parameters, whereas Gemini Advanced showed significant differences across only two. ChatGPT-4o appeared to integrate assessment data more effectively, but both models remained limited in personalization.	Recommendations remained constrained and were not tested as delivered patient interventions.	OA publication support from the Scientific and Technological Research Council of Türkiye (TÜBİTAK)
Gürses et al. (2025b) [44]	Rehabilitation planning / prescription	Compare personalized rehabilitation recommendations from LLMs with physiotherapist consensus across 50 program parameters.	Agreement with physiotherapist recommendations across program parameters	Overall agreement with the physiotherapist consensus was approximately 74% for ChatGPT-4o and 70% for Gemini Advanced. Exercise frequency, sets, and progression/specificity remained key areas of mismatch.	Substantial agreement did not eliminate clinically relevant differences in prescription details, so expert supervision remained necessary.	None
Hao et al. (2025) [45]	Education / simulation / feedback	Evaluate ChatGPT's clinical reasoning through the sequential Subjective, Objective, Assessment, and Plan tasks.	Accuracy and completeness by SOAP domain	Mean accuracy scores were 4.3 (Subjective), 3.7 (Objective), 4.4 (Assessment), and 4.3 (Plan); completeness scores were 2.4, 2.0, 2.8, and 2.4. Objective examination planning was weakest, with generic test lists, incomplete red-flag screening, and limited stage-specific detail.	Weaknesses in objective examination/red-flag screening are clinically significant; educational use requires supervision.	None
Inoue et al. (2025) [46]	Rehabilitation planning / prescription	Generate occupational therapy programs for five stroke cases	Feasibility and professional adequacy of generated programs	All evaluated scores were 2/4 or lower, and inter-rater agreement was low. Outputs	Poor specificity and individualization argue against	None

Study	Primary application	Task evaluated	Main outcome / measure	Main finding	Safety / clinical limitation	Funding/financial support
		with psychological symptoms using ChatGPT/GPT (exact version not reported in the verified source) and assess feasibility and specialization.		were described as generic and insufficiently specific, individualized, and specialized for OT practice.	independent clinical use.	
Lee & Park (2025) [47]	Education / simulation / feedback	Generate neurological OT case studies and evaluate their clinical realism, comprehensiveness, and educational value.	Clinical realism, comprehensiveness and educational value	Mean ratings were 4.22/5 for clinical realism, 4.56/5 for comprehensiveness, and 4.44/5 for educational value.	Expert-rated case quality was high, but actual student learning effectiveness and clinical transfer were not assessed.	National Research Foundation of Korea, funded by the Ministry of Education (2021R1I1A3041487); Paan Scholarship and Culture Foundation; Soonchunhyang University Research Fund
Marcaccini et al. (2025) [48]	Rehabilitation planning / prescription	Generate personalized rehabilitation programs for surgical scenarios and compare model outputs.	Clinical relevance, accuracy/readability and comparative performance	ChatGPT-4o had the highest reported clinical-relevance score (4.90±0.32), followed by DeepSeek V3 (4.00±0.82), Gemini 2 (3.90±0.74), and Copilot (2.70±0.82). Differences among models were significant (p<.001). Gemini 2 produced the most readable outputs.	Scenario-based expert rating; clinical effectiveness and patient safety require real-world validation.	None
Mykhalko et al. (2025) [49]	Rehabilitation planning / prescription	Generate individualized one-month physical rehabilitation plans and evaluate six clinical criteria.	Individualization, clinical accuracy, safety, progressive design, feasibility/accessibility, focus on result; overall suitability	The overall mean score was 4.30±0.28; clinical accuracy (4.59±0.59) and safety (4.41±0.71) were the highest. 72% of plans were "Suitable for use with corrections"; none were completely unsuitable. ICC = .80–.86 and weighted κ = .77.	Most plans required expert correction; authors concluded they were not ready for use without expert review or modification.	NR
Okuno et al. (2025) [50]	Education / simulation / feedback	Implement an LLM-assisted, story-centered curriculum and compare its learning outcomes with those of a historical cohort.	Pre/post knowledge score change and student experience	Score change was 105 (IQR 78-140) in the story-centered group vs 80 (50-110) in controls (p<.001), and posttest scores were 195 vs 180 (p=.013). More than 90% of students reported positive experiences.	Historical control design is susceptible to temporal/cohort confounding; educational outcomes do not demonstrate clinical benefit for patients.	None
Rossettini et al. (2025) [51]	Guideline-concordant clinical support	Compare six chatbot recommendations with CPG	CPG match rate and Cohen's kappa	Match rates were 33% for ChatGPT-3.5, ChatGPT-4o, and Claude; 44% for Copilot; 63% for	Authors discouraged the adoption of these AI chatbots as	Italian Ministry of Health; OA publication support from the Department of

Study	Primary application	Task evaluated	Main outcome / measure	Main finding	Safety / clinical limitation	Funding/financial support
		recommendations.		Gemini; and 67% for Perplexity. Corresponding Cohen κ values were .13, .11, .16, .22, .38, and .49.	patient information tools for lumbosacral radicular pain due to insufficient guideline agreement.	Innovation, Research, University and Museums of the Autonomous Province of Bolzano/Bozen
Safran & Yildirim (2025) [52]	Guideline-concordant clinical support	Evaluate ChatGPT responses for clarity, accuracy, relevance, completeness, consistency, and alignment with CPG.	Five quality dimensions and weighted kappa for guideline alignment	Mean scores were clarity 4.85, accuracy 4.62, relevance 4.50, completeness 4.20, and consistency 3.85. Weighted κ was .90 for disease-information items and .56 for rehabilitation items.	The rehabilitation-specific agreement was only moderately effective; its use is best considered supplementary.	None
Saglam et al. (2025) [53]	Clinical reasoning / decision support	Compare GPT-4 and GPT-3.5 in clinical decision-making, treatment planning, and rehabilitation protocols.	Comparative clinical-decision scores and internal consistency	GPT-4 significantly outperformed GPT-3.5 across criteria (overall $t(55) = 10.45$, $p < .001$), including treatment planning ($F(1,55) = 35.22$) and rehabilitation protocols ($F = 32.10$, $p < .001$).	Better relative performance does not eliminate the need for professional verification; evaluation is only scenario-based.	None
Sudo et al. (2025) [54]	Education / simulation / feedback	Compare zero-shot and few-shot AI-generated feedback on student case reports and examine perceptions.	Feedback preference/usefulness, fairness, affect, and qualitative perceptions	26/35 students (74%) preferred few-shot feedback overall; the total feedback perception questionnaire score did not differ significantly ($p=.22$), although several fairness and usefulness items favored few-shot prompting.	Prompt strategy and disclosure of AI identity influenced perceptions; educational feedback should be teacher-supervised.	None
Zhang et al. (2025) [55]	Guideline-concordant clinical support	Compare ChatGPT-4o and ChatGPT-4 Turbo recommendations against evidence-based CPGs.	Accuracy, consistency, self-awareness, and fabrication/falsification dimensions	Both models showed similar overall performance: accuracy about 4.1/5, consistency about 4.6/5, self-awareness 4.3-4.5, and fabrication/falsification 4.5-4.6, with no significant overall differences. Performance was stronger on closed-ended questions and positive recommendations.	Good average ratings do not ensure full adherence to guidelines; expert verification remains necessary.	None
Zou et al. (2025) [56]	Clinical reasoning / decision support	Compare triage classifications from a DRP tool, ChatGPT-4 (via a Chinese network portal), and rehabilitation doctors.	Pairwise kappa, ICC, and exact consistency	The Tool-ChatGPT agreement was higher (κ = .602; ICC = .669; exact consistency 63.67%) than Tool-Doctor (κ = .298; ICC = .344; 40.33%) or ChatGPT-Doctor	ChatGPT/tool classifications differed substantially from those of doctors; agreement does not equate to patient-	Shenzhen Sanming Project of Medicine (SZSM202111010); Shenzhen Health Economics Society Scientific Research Fund Project (2025343)

Study	Primary application	Task evaluated	Main outcome / measure	Main finding	Safety / clinical limitation	Funding/financial support
				(κ = .311; ICC = .397; 40.0%).	outcome validity.	
Bilika et al. (2024) [57]	Clinical reasoning / decision support	Assess the consistency and accuracy of physiotherapy assessment responses and differential diagnostic reasoning.	Consistency, omissions and differential-diagnosis reasoning	Responses varied in completeness; reassessment and subjective examination were omitted in 30% and 40% of responses, respectively. Reasoning was more convincing in the simple case, and question phrasing materially affected the answers.	Inconsistent omissions and prompt sensitivity pose problems for unsupervised clinical reasoning.	None
Mansour & Wong (2024) [58]	Education / simulation / feedback	Use generative AI in a fieldwork-readiness assignment and assess students' comfort and confidence.	Comfort and confidence using ChatGPT for intervention ideas	Comfort using ChatGPT for intervention ideas increased significantly ($p<.001$). Confidence in generating occupation- and exercise-based interventions increased numerically but did not reach conventional significance ($p=.066$ and .071).	Single-group design with attrition and no direct assessment of clinical competence or patient outcomes.	None
Sawamura et al. (2024) [59]	Guideline-concordant clinical support	Ask 15 guideline-derived Japanese clinical questions (5 each from stroke, musculoskeletal disorders, and internal disorders) and request supporting references (PMID/DOI).	Response-content accuracy and reference existence/appropriateness	Clinical answer content was often reasonable, but reference accuracy was poor, with nonexistent or misinterpreted citations.	Citation hallucinations or incorrect references can directly undermine evidence-based decision support.	Heisei College of Health Sciences President-Recommended Research Activity Grant (2023)
Zhenzhu et al. (2024) [60]	Adaptive feedback / rehabilitation support	Compare direct GPT-4 responses with guideline-based GPT agents on TBI rehabilitation questions.	Responsiveness, explainability, and answer quality	Guideline-based GPT agents improved responsiveness, explainability, and accuracy relative to direct GPT-4 on the study question set.	Non-human benchmark; generalizability to real patients and evaluator independence remain limited.	Ningbo Leading Medical and Health Discipline (2022-S02); Hwa Mei Medical Education Research Project (2021HMJYZD05); Ningbo No. 2 Hospital HwaMei Research Foundation (2024HMZD16 and 2023HMZD03); Ningbo Health and Technology Plan (2022Y10 and 2022Y11); Ningbo Major Science and Technology Project (2022Z126)

Study	Primary application	Task evaluated	Main outcome / measure	Main finding	Safety / clinical limitation	Funding/financial support
Zhang et al. (2023) [61]	Clinical reasoning / decision support	Generate rehabilitation prescriptions and ICF coding for a textbook stroke case, then compare with textbook/reference judgment.	Clinical appropriateness of rehabilitation recommendations and ICF coding	ChatGPT-4 generated broad rehabilitation prescriptions and largely appropriate ICF codes, but the authors reported an error in interpreting an ICF category.	Single test case only; useful for hypothesis generation, not for clinical validation.	Kyorin University School of Medicine Education and Research Fund

ADL, activities of daily living; AI, artificial intelligence; CPG, clinical practice guideline; DRP, diagnosis–rehabilitation pathway/tool as reported by the source; EMG, electromyography; FPQ, Feedback Perceptions Questionnaire; ICC, intraclass correlation coefficient; ICF, International Classification of Functioning, Disability and Health; IQR, interquartile range; LLM, large language model; OA, osteoarthritis; OT, occupational therapy; PBL, problem-based learning; PT, physical therapy; TBI, traumatic brain injury; NR, not reported.

Supplementary Table S6. Operational coding framework and ambiguity-resolution rules for the six application domains.

Final domain	Initial/working codes	Primary assignment rule	Tie-break rule
Clinical reasoning and decision support	Reasoning; diagnosis; examination planning; triage; treatment decision	Clinical reasoning or decision quality is the primary outcome.	Plan-generating studies remain here only when reasoning, rather than the plan itself, is evaluated.
Rehabilitation education, simulation, and feedback	Education; PBL; simulation; curriculum; formative feedback; learner outcomes	Teaching, simulation, feedback, or learner outcomes are the primary focus.	Educational cases remain here unless clinical decision-support performance is evaluated.
Rehabilitation planning and prescription	Rehabilitation plan; exercise prescription; program generation; personalization	Rehabilitation plans, prescriptions, progression, personalization, or feasibility are generated or evaluated.	Assign to guideline support when CPG concordance is the primary outcome.
Guideline adherence and clinical-question support	CPG concordance; guideline alignment; evidence-based Q&A	AI responses are primarily compared with CPGs or reference standards.	Prioritize this domain when concordance is the primary outcome.
Adaptive feedback and rehabilitation support	Adaptive support; therapist-facing tools; agents; guided delivery	AI supports feedback, intervention delivery, implementation, or rehabilitation workflows.	Include single-case implementations when evaluating intervention outcomes.
Assessment and functional classification	Assessment; multimodal classification; functional or neuromuscular state	The primary objective is the assessment, classification, scoring, or interpretation of functional or multimodal data.	Retain this domain when assessment or classification remains primary despite subsequent recommendations.

CPG, clinical practice guideline; PBL, problem-based learning.

Supplementary Table S7. Preferred Reporting Items for Systematic reviews and Meta-Analyses extension for Scoping Reviews (PRISMA-ScR) Checklist

SECTION	ITEM	PRISMA-ScR CHECKLIST ITEM	REPORTED ON PAGE #
**TITLE**			
Title	1	Identify the report as a scoping review.	p. 1
**ABSTRACT**			
Structured summary	2	Provide a structured summary that includes (as applicable): background, objectives, eligibility criteria, sources of evidence, charting methods, results, and conclusions that relate to the review questions and objectives.	p. 1
**INTRODUCTION**			
Rationale	3	Describe the rationale for the review in the context of what is already known. Explain	pp. 1–2

SECTION	ITEM	PRISMA-ScR CHECKLIST ITEM	REPORTED ON PAGE #
		why the review questions/objectives lend themselves to a scoping review approach.	
Objectives	4	Provide an explicit statement of the questions and objectives being addressed with reference to their key elements (e.g., population or participants, concepts, and context) or other relevant key elements used to conceptualize the review questions and/or objectives.	p. 2
**METHODS**			
Protocol and registration	5	Indicate whether a review protocol exists; state if and where it can be accessed (e.g., a Web address); and if available, provide registration information, including the registration number.	p. 3
Eligibility criteria	6	Specify characteristics of the sources of evidence used as eligibility criteria (e.g., years considered, language, and publication status), and provide a rationale.	pp. 3–4; Table 1
Information sources*	7	Describe all information sources in the search (e.g., databases with dates of coverage and contact with authors to identify additional sources), as well as the date the most recent search was executed.	pp. 4–5; Supplementary Table S1, pp. 25–26
Search	8	Present the full electronic search strategy for at least 1 database, including any limits used, such that it could be repeated.	p. 5; Table 2, p. 5; Supplementary Table S1, pp. 25–26
Selection of sources of evidence†	9	State the process for selecting sources of evidence (i.e., screening and eligibility) included in the scoping review.	p. 5
Data charting process‡	10	Describe the methods of charting data from the included sources of evidence (e.g., calibrated forms or forms that have been tested by the team before their use, and whether data charting was done independently or in duplicate) and any processes for obtaining and confirming data from investigators.	pp. 5–6
Data items	11	List and define all variables for which data were sought and any assumptions and simplifications made.	p. 6
Critical appraisal of individual sources of evidence§	12	If done, provide a rationale for conducting a critical appraisal of included sources of evidence; describe the methods used and how this information was used in any data synthesis (if appropriate).	pp. 6–7; Table 3, p. 9; Supplementary Table S2, pp. 26–30
Synthesis of results	13	Describe the methods of handling and summarizing the data that were charted.	p. 7; Supplementary Table S6, p. 50
**RESULTS**			
Selection of sources of evidence	14	Give numbers of sources of evidence screened, assessed for eligibility, and included in the review, with reasons for exclusions at each stage, ideally using a flow diagram.	pp. 7–9; Figure 1, pp. 8–9
Characteristics of sources of evidence	15	For each source of evidence, present characteristics for which data were charted and provide the citations.	pp. 9–13; Supplementary Table S3, pp. 31–33
Critical appraisal within sources of evidence	16	If done, present data on critical appraisal of included sources of evidence (see item 12).	p. 9; Table 3, p. 9; Supplementary Table S2, pp. 26–30
Results of individual sources of evidence	17	For each included source of evidence, present the relevant data that were charted that relate to the review questions and objectives.	pp. 9–15; Supplementary Tables S3–S5, pp. 31–49

SECTION	ITEM	PRISMA-ScR CHECKLIST ITEM	REPORTED ON PAGE #
Synthesis of results	18	Summarize and/or present the charting results as they relate to the review questions and objectives.	pp. 9–16; Tables 4–6, pp. 12–15; Figures 2–5, pp. 10–16
**DISCUSSION**			
Summary of evidence	19	Summarize the main results (including an overview of concepts, themes, and types of evidence available), link to the review questions and objectives, and consider the relevance to key groups.	pp. 16–20
Limitations	20	Discuss the limitations of the scoping review process.	p. 19
Conclusions	21	Provide a general interpretation of the results with respect to the review questions and objectives, as well as potential implications and/or next steps.	p. 20
**FUNDING**			
Funding	22	Describe sources of funding for the included sources of evidence, as well as sources of funding for the scoping review. Describe the role of the funders of the scoping review.	p. 15; p. 20; Supplementary Table S5, pp . 41–50

JBI = Joanna Briggs Institute; PRISMA-ScR = Preferred Reporting Items for Systematic reviews and Meta-Analyses extension for Scoping Reviews.

* Where *sources of evidence* (see second footnote) are compiled from, such as bibliographic databases, social media platforms, and Web sites.

† A more inclusive/heterogeneous term used to account for the different types of evidence or data sources (e.g., quantitative and/or qualitative research, expert opinion, and policy documents) that may be eligible in a scoping review as opposed to only studies. This is not to be confused with *information sources* (see first footnote).

‡ The frameworks by Arksey and O'Malley (6) and Levac and colleagues (7) and the JBI guidance (4, 5) refer to the process of data extraction in a scoping review as data charting.

§ The process of systematically examining research evidence to assess its validity, results, and relevance before using it to inform a decision. This term is used for items 12 and 19 instead of "risk of bias" (which is more applicable to systematic reviews of interventions) to include and acknowledge the various sources of evidence that may be used in a scoping review (e.g., quantitative and/or qualitative research, expert opinion, and policy document).
